# Human Cytomegalovirus Fcγ Binding Proteins gp34 and gp68 Antagonize Fcγ Receptors I, II and III

**DOI:** 10.1371/journal.ppat.1004131

**Published:** 2014-05-15

**Authors:** Eugenia Corrales-Aguilar, Mirko Trilling, Katja Hunold, Manuela Fiedler, Vu Thuy Khanh Le, Henrike Reinhard, Katrin Ehrhardt, Eva Mercé-Maldonado, Enver Aliyev, Albert Zimmermann, David C. Johnson, Hartmut Hengel

**Affiliations:** 1 Institute for Virology, Heinrich-Heine-University Düsseldorf, Düsseldorf, Germany; 2 Institute for Virology, University Hospital Essen, University of Duisburg-Essen, Essen, Germany; 3 Institute of Virology, University Medical Center, Albert-Ludwigs-University Freiburg, Freiburg, Germany; 4 Department of Molecular Microbiology & Immunology, Oregon Health Sciences University, Portland, Oregon, United States of America; University of Cambridge, United Kingdom

## Abstract

Human cytomegalovirus (HCMV) establishes lifelong infection with recurrent episodes of virus production and shedding despite the presence of adaptive immunological memory responses including HCMV immune immunoglobulin G (IgG). Very little is known how HCMV evades from humoral and cellular IgG-dependent immune responses, the latter being executed by cells expressing surface receptors for the Fc domain of IgG (FcγRs). Remarkably, HCMV expresses the *RL11*-encoded gp34 and *UL119-118*-encoded gp68 type I transmembrane glycoproteins which bind Fcγ with nanomolar affinity. Using a newly developed FcγR activation assay, we tested if the HCMV-encoded Fcγ binding proteins (HCMV FcγRs) interfere with individual host FcγRs. In absence of gp34 or/and gp68, HCMV elicited a much stronger activation of FcγRIIIA/CD16, FcγRIIA/CD32A and FcγRI/CD64 by polyclonal HCMV-immune IgG as compared to wildtype HCMV. gp34 and gp68 co-expression culminates in the late phase of HCMV replication coinciding with the emergence of surface HCMV antigens triggering FcγRIII/CD16 responses by polyclonal HCMV-immune IgG. The gp34- and gp68-dependent inhibition of HCMV immune IgG was fully reproduced when testing the activation of primary human NK cells. Their broad antagonistic function towards FcγRIIIA, FcγRIIA and FcγRI activation was also recapitulated in a gain-of-function approach based on humanized monoclonal antibodies (trastuzumab, rituximab) and isotypes of different IgG subclasses. Surface immune-precipitation showed that both HCMV-encoded Fcγ binding proteins have the capacity to bind trastuzumab antibody-HER2 antigen complexes demonstrating simultaneous linkage of immune IgG with antigen and the HCMV inhibitors on the plasma membrane. Our studies reveal a novel strategy by which viral FcγRs can compete for immune complexes against various Fc receptors on immune cells, dampening their activation and antiviral immunity.

## Author Summary

Herpes viruses persist lifelong continuously alternating between latency and virus production and transmission. The latter events occur despite the presence of immune IgG antibodies. IgG acts by neutralization of virions and activation of immune cells bearing one or more surface receptors, called FcγRs, recognizing the constant Fc domain of IgG. Activating FcγRs induce a wide range of immune responses, including antibody dependent cellular cytotoxicity (ADCC) of virus-infected cells by natural killer (NK) cells, cytokine secretion and the uptake of immune complexes to enhance antigen presentation to T cells. We demonstrate that the HCMV glycoproteins *RL11*/gp34 and *UL119-118*/gp68 block IgG-mediated activation of FcγRs. A novel reporter cell-based assay was used to test FcγRs individually and assess their relative susceptibility to each antagonist. This approach revealed that gp34 and gp68 block triggering of activating FcγRs, i.e. FcγRI (CD64), FcγRII (CD32A) and FcγRIII (CD16). Co-immunoprecipitation showed the formation of ternary complexes containing IgG, IgG-bound antigen and the viral antagonists on the cell surface. Assigning the redundant abilities of HCMV to hinder IgG effector responses to the viral Fc binding proteins, we discuss gp34 and gp68 as potential culprits which might contribute to the limited efficacy of therapeutic IgG against HCMV.

## Introduction

Human cytomegalovirus (HCMV) constitutes the prototypical human pathogenic β-herpesvirus found worldwide with high immunoglobulin G (IgG) seroprevalence rates of 50–98% [1]. Despite the expression of a very large antigenic proteome of approximately 750 translational products [2], HCMV avoids sterile immunity and invariably persists lifelong in the human host in a latent state with periodic phases of reactivation and virus shedding. While infection of immune competent individuals is usually subclinical, HCMV causes severe symptoms in immunocompromised individuals and congenitally infected newborns [1], [3]. Cytomegalovirus immune control is organized in a hierarchical as well as redundant manner, with crucial roles for natural killer (NK) cells as well as T lymphocytes [4]. HCMV expresses a large set of immune evasion genes that impair recognition of infected cells by CD8+, CD4+ and NK effector cells and thus facilitate virus persistence, spread and superinfection [5]–[7] while cellular immune responses are nevertheless indispensable for CMV immune surveillance. Experimental and clinical evidence suggest that cytomegalovirus can persist for the lifetime by effectively defending itself from both cellular and humoral immunity. In the absence of either viral immune evasion genes or subsets of immune cells, the balance of pathogenesis versus clearance of the virus can be tilted. For example, B cell deficient mice exhibit a much higher susceptibility during recurrent mouse cytomegalovirus (MCMV) infection compared to control mice, reflected by 100–1,000-fold increased titers in the absence of CMV-specific IgG [8]. Moreover, adoptive transfer of memory B cells into naïve Rag^−/−^ mice is sufficient for long term protection from lethal MCMV disease [9], and passive immunization with immune IgG reduces MCMV-induced pathology in newborn mice [10]. In clinical settings, HCMV-immune IgG preparations are used with varying degrees of success. Human intravenous hyperimmune immunoglobulin against HCMV (e.g. Cytotect) significantly lowers the risk of congenital CMV infection and disease at birth when given to primary HCMV-infected pregnant women [11]. Nevertheless, meta-analyses of clinical studies with solid organ transplant recipients as well as patients undergoing hematopoietic stem cell transplantation document little if any benefit of IgG prophylaxis against HCMV disease [12]–[14].

IgG antibodies have two functional domains: the fragment antigen binding (Fab) that contains the paratope recognizing the respective epitope of the antigen and the fragment crystallisable (Fc) which recruits IgG effector functions. Receptors for the Fc domain of IgG (FcγRs) are expressed on immune cells to connect the humoral and cellular branches of immunity. Upon IgG binding and receptor activation, FcγRs trigger a diversity of effector responses including antibody-dependent cellular cytotoxicity (ADCC), phagocytosis, endocytosis of immune complexes and cytokine production. Importantly, the set of human FcγRs includes different activating members, i.e. FcγRI (CD64), FcγRIIA (CD32A), FcγRIIC (CD32C) and FcγRIIIA (CD16) which differ in immune cell distribution, affinity for distinct IgG subclasses [15] and effector functions elicited upon activation [16]–[19].

Fcγ binding activity on the surface of HCMV-infected cells has long been reported [20], but the consequences of Fcγ binding to immune responses are unknown [21]. The HCMV Fcγ binding proteins gp34 and gp68 are type I transmembrane glycoproteins encoded by independent genes, *RL11* and *UL119-UL118*, respectively, which are fully dispensable for HCMV replication *in vitro* [22], [23]. Both HCMV proteins show cell surface disposition and exquisite ligand specificity for human IgG but no other Ig classes (e.g. IgA or IgM) [22]. Minimal sequence relatedness in their extracellular domains with particular immunoglobulin supergene family domains present in FcγRI and FcγRII/III suggests differing binding characteristics from those of host FcγRs [22]. In contrast to host FcγRs, both gp34 and gp68 recognize Fcγ in a manner independent of N-linked glycosylation, further corroborating a binding mode to Fcγ which is distinguishable from host FcγRs [24].

Cytomegaloviruses frequently reactivate and can super-infect despite the presence of relatively high levels of HCMV-specific IgG [25]–[27] which raises the apparent question by which mechanism HCMV avoids antibody-mediated immune control. One conceivable possibility is that HCMV-encoded FcγRs gp34 and gp68 compete with cellular FcγRs. To test this hypothesis, we had to established a suitable FcγR activation assay that allows a comprehensive analysis of the activation status of individual FcγRs [28]. We compared cells infected with viruses lacking gp34 and/or gp68 upon opsonization with graded doses of polyclonal HCMV-immune IgG. As a control, we included the human α-herpesvirus herpes simplex virus 1 (HSV-1), which expresses a viral FcγR composed of the glycoproteins gE and gI that is known to protect infected cells from ADCC elicited by HSV-immune IgG [29], [30]. Our approach identifies FcγRI, FcγRIIA and FcγRIII as principal targets of both HCMV gp34 and gp68, while the prototypic HSV-1 FcγR gE was found to inhibit only FcγRIIA and FcγRIII. This is the first experimental proof of CMV-encoded glycoproteins interfering with IgG-mediated immunity.

## Results

### HCMV and HSV-1 encoded viral FcγRs (vFcγRs) bind Fcγ on the surface of infected cells

**Figure 1:**
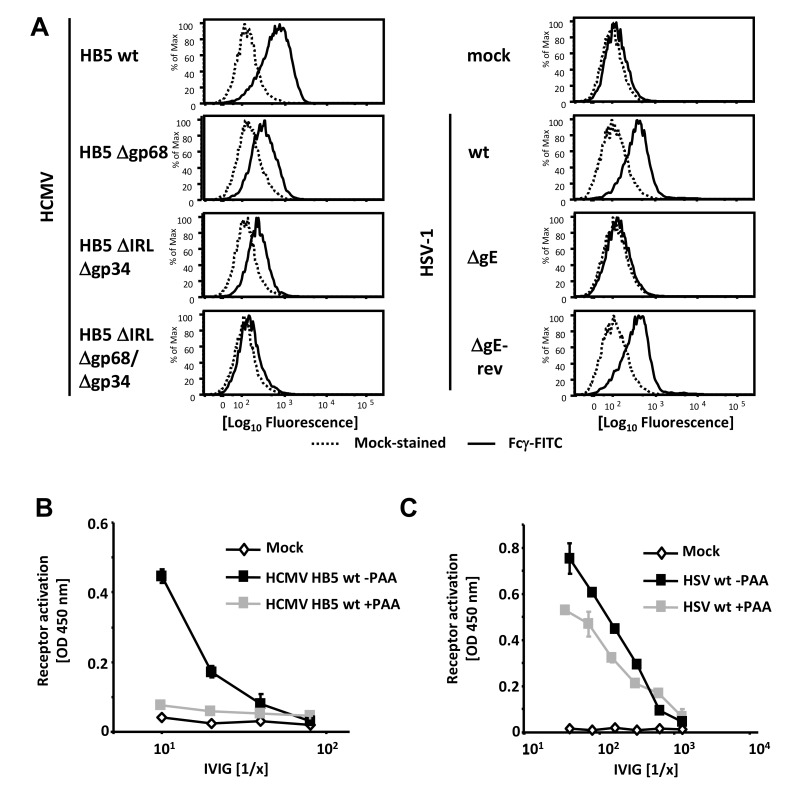
Viral FcγRs bind Fcγ on the cell surface and opsonizing IgG dependent FcγR activation is restricted to the late phase of HCMV but not HSV replication. (**A**) MRC-5 cells were infected with 2 PFU/cell for 72 hpi with HCMV HB5 wildtype, HB5Δgp68, HB5ΔIRLΔgp34 or HB5ΔIRLΔgp68/Δgp34 (left) or 24 hpi with HSV-1 (right). Cells were resuspended with PBS/2% (vol/vol) FCS containing 2 mM EDTA, stained with hFcγ-FITC and measured in a FACS Canto II. Dead cells were excluded by PI or DAPI-staining. (**B**) MRC-5 cells were infected with 2–3 PFU/cell of HCMV HB5 wt or (**C**) HSV-1 strain F. After centrifugation enhancement of infection, cells were incubated 3 h at 37°C at 5% CO_2_. After washing once, PAA (250 µg/ml) in D-MEM 10% (vol/vol) FCS was added. 48 hpi for HSV or 72 hpi for HCMV, cells were opsonized with grading dilutions of Cytotect for 30 minutes, washed twice with D-MEM 10% (vol/vol) FCS and co-cultivated with 1×10^5^ BW:FcγR-ζ reporter cells per well. Measurement of mIL-2 in supernatants after 16 h of co-cultivation of reporter cells with targets was performed by ELISA. Values are presented in the graphic as OD 450 nm. 3 independent wells were measured, means are shown with standard deviations (error bars) for 2 independent experiments. Significance of results (Student's t-test) is presented in Table S1 as *: p<0.05 **: p<0.01 ***: p<0.001.

To assess the relative surface density of viral Fcγ receptors on the plasma membrane of HSV and HCMV-infected cells, Fcγ binding was evaluated by flow cytometry using FITC-labeled Fcγ fragment. As expected, Fcγ-FITC surface binding was observed for HSV wt virus- and the gE revertant virus-infected cells, but not for cells infected with ΔgE HSV **(** **Figure 1A** **)**. MRC-5 cells infected with either of two HCMV HB5 single vFcγR deletion mutants, HB5Δgp68 or HB5ΔIRLΔgp34 [22], were decorated at the cell surface with diminished levels of Fcγ-FITC compared to wt-infected control cells **(** **Figure 1A** **)**. Cells infected with a HCMV mutant lacking both gp68 and gp34 [31] showed only very low Fc-binding when compared to mock-infected fibroblasts **(** **Figure 1A** **)**. Together with our previous experiments documenting Fcγ binding upon ectopic expression of gp34 and gp68 using recombinant vaccinia viruses (rVACV) [22] these data define gp34 and gp68 to be sufficient and essential for Fc binding by HCMV-infected cells.

### FcγR activating IgG almost exclusively recognizes antigens expressed during the late phase of HCMV replication

In productively infected cells, herpesvirus gene expression is regulated in a cascade fashion. Viral proteins encoded by genes of the early phase of infection are required for viral DNA replication, which is a prerequisite for the subsequent expression of structural virion proteins during the late phase of gene expression. To assign the immune-dominant HCMV and HSV surface antigens recognized by opsonizing IgG to the temporal class of genes, we applied the novel reporter cell system allowing quantification of host FcγR activation [28]. This assay is based on co-cultivation of antigen-bearing cells with reporter cells stably expressing FcγR-ζ chain chimeric receptors which produce mouse IL-2 upon recognition of immune IgG, provided that the opsonizing antibody is able to activate the particular FcγR [28]. Importantly, BW5147:FcγR-ζ reporter cells are neither activated by cells lacking the appropriate antigen (e.g., non-infected cells) nor by antigen-expressing cells which had been cultivated in absence of IgG or in presence of non-immune IgG, proving strict antigen- and immune IgG specificity of the assay (**Figure S1** and Reference [28]). Late phase gene expression was blocked using 250 µg/ml phosphonoacetic acid (PAA) which blocks the viral DNA polymerase and is the active component of the clinically approved anti-HCMV drug Foscarnet. At 72 and 48 hpi, resp., infected cells were opsonized with graded concentrations of intravenous immunoglobulin (IVIG) Cytotect as a source of human HCMV- and HSV-immune IgG. As expected, immune IgG did not induce receptor activation (IL-2 response) in the presence of mock-infected cells **(** **Figure 1B** **)**. While late antigens of HCMV efficiently triggered FcγRIII reporter cells, infected cells arrested in the early phase of replication elicited very poor if any responses **(** **Figure 1B** **)**. On the contrary, early antigens of HSV-1 were sufficient to efficiently trigger FcγRIII and HSV late antigens only slightly increased FcγR-ζ activation **(** **Figure 1C** **)**. Despite the fact that Cytotect is prepared from donors selected for particularly high HCMV IgG titers [11], [32] we found - in agreement with earlier studies [28] - that Cytotect contains higher titers of HSV-immune IgG activating FcγRIII compared to FcγRIII-reactive HCMV-immune IgG. Due to this fact, IVIG dilutions used for HSV-1 and HCMV experiments had to be chosen differently. The relatively poor responses triggered by IgG opsonized HCMV-infected cells was compatible with our hypothesis that HCMV could be able to reduce the activation of FcγRIII by immune IgG, and that vFcγR gp68 and gp34 might represent candidates for such inhibition.

### Interference with host FcγR activation by HSV-1 gE, HCMV gp68 and HCMV gp34

**Figure 2:**
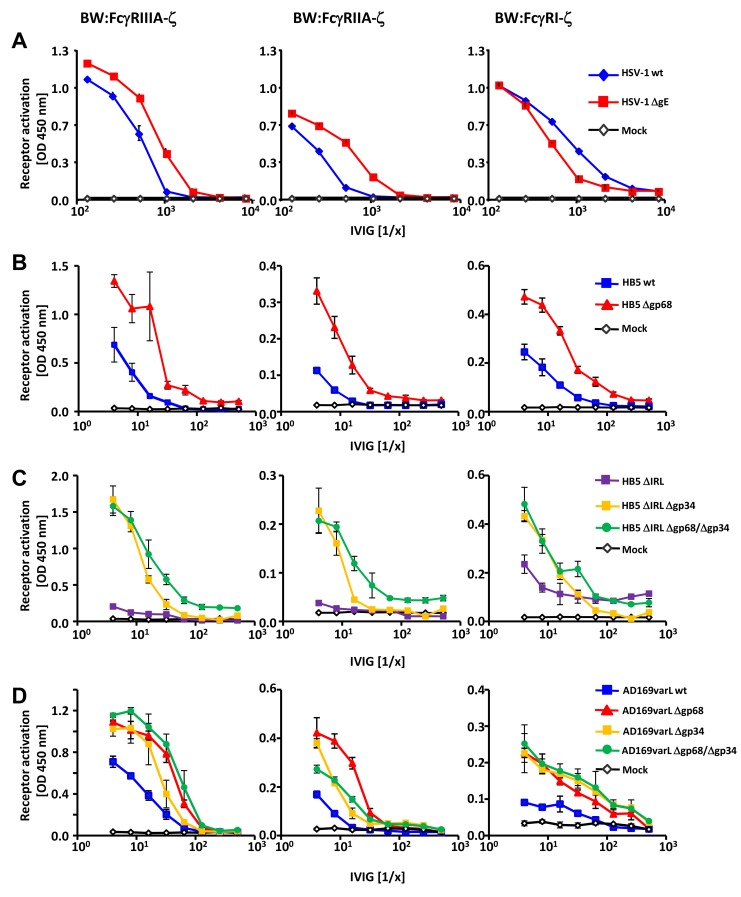
HSV-1 gE, HCMV gp68 and HCMV gp34 interfere with host FcγR activation upon opsonization of cells with polyclonal immune IgG. (**A**) The HSV vFcγR gE inhibits FcγRIIIA and FcγRIIA activation but fails to inhibit FcγRI. Human MRC-5 fibroblasts were infected with 2 PFU/cell of HSV-1 strain F wt and ΔgE for 24 h. Cells were opsonized with Cytotect at different concentrations for 30 min. After removing of unbound antibodies with D-MEM 10% (vol/vol) FCS, 1×10^5^ BW:FcγR-ζ transfectants per well were added and co-cultivated overnight. BW:FcγR activation was determined by measuring mIL-2 by ELISA. Three independent replicates were measured; means with standard deviations (error bars) are shown for 4 independent experiments. (**B**) HCMV vFcγR gp68 interferes with FcγRIIIA, FcγRIIA and FcγRI activation. MRC-5 cells were infected with HCMV HB5 wt virus or HB5Δgp68 (2 PFU/cell) for 72 h. Fibroblasts were opsonized with Cytotect at different concentrations for 30 min. After removing of unbound antibodies by washing, 1×10^5^ BW:FcγR-ζ transfectants were added per well. Measurement of mIL-2 in supernatants after 16 h of co-cultivation of reporter cells with targets was performed by ELISA. Values are presented in the graphic as OD 450 nm. Three independent wells were measured; means with standard deviations (error bars) are shown for 4 independent experiments. Significance of results (Student's t-test) are presented in Table S1 as *: p<0.05 **: p<0.01 ***: p<0.001. (**C**) HCMV vFcγR gp34 interferes with FcγRIIIA, FcγRIIA and FcγRI activation. As in (B) but MRC-5 cells were infected with HCMV HB5ΔIRL, HB5ΔIRLΔgp34 or HB5ΔIRLΔgp68/Δgp34 (2 PFU/cell) for 72 h. (**D**) gp34 and gp68 interfere with FcγR activation in AD169varL infected cells. As in (B) but MRC-5 cells were infected with AD169varL wt, AD169varLΔgp68, AD169varLΔgp34 or AD169varLΔgp68/Δgp34.

To test the conjecture that vFcγR gp68 and gp34 could prevent host FcγR activation by HCMV IgG, we pursued the BW5147:FcγR-ζ reporter cell approach. To assess if and to what degree viral FcγRs can interfere with host FcγRs activation, we first compared responses of FcγRIII reporter cells co-cultured with IgG-opsonized HSV-1 wt infected vs. HSV-1 ΔgE-infected MRC-5 cells **(** **Figure 2A** **)**. Inhibition of ADCC by PBMCs was reported to be a function of the prototypic HSV-1 FcγR gE [30], albeit the specific host FcγRs which are blocked by gE have not been elucidated yet. As before **(** **Figure 1C** **)**, we observed a dose-dependent activation of the reporter cells upon co-cultivation with HSV-1-infected cells but not with mock infected cells. The ΔgE HSV-1 mutant led to an increased activation of FcγRIIIA upon opsonization of target cells with Cytotect, in accordance with published data [29], [30]. The same type of result was obtained when using BW5147:FcγRIIA-ζ reporter cells, indicating that gE also antagonizes activation of this host Fcγ receptor. Surprisingly, gE enhanced, rather than inhibited the IgG-dependent activation as deduced from the overall superior activation of the chimeric FcγRI-ζ by wt HSV-1 when compared to HSV-1 ΔgE **(** **Figure 2A** **)**.

The results of HSV-1 gE encouraged us to subsequently test HCMV HB5Δgp68 and HB5ΔIRLΔgp34 mutants using the same experimental strategy. MRC-5 fibroblasts were left uninfected or infected with wt-HCMV strain HB5 versus HB5Δgp68 **(** **Figure 2B** **)**, or with HB5ΔIRL (the parental virus of the following mutants) versus HB5ΔIRLΔgp34 and HB5ΔIRLΔgp68/Δgp34, resp., a double mutant lacking both gp68 and gp34 **(** **Figure 2C** **)**. 72 h post HCMV infection, target cells were incubated with graded dilutions of Cytotect before BW:FcγRIIIA-ζ responder cells were added. HB5Δgp68 induced a clearly higher response over an extended range of IgG dilutions compared to wt-HCMV HB5 infected cells. Likewise, HB5ΔIRLΔgp34-opsonized cells induced clearly higher reporter cell responses compared with HB5ΔIRL opsonized targets, while HB5ΔIRLΔgp68/Δgp34 exhibited only marginal further increase of the response (**Figure 2C**). These results suggested that cells infected with virus mutants lacking viral Fc-binding proteins elicit exaggerated activation of FcγRIIIA, provided that the amount of opsonizable HCMV antigens is indeed comparable between the viruses analyzed. To verify this supposition, cells were labelled with F(ab)_2_ antibody fragments prepared from Cytotect and analysed by FACS. As shown in **Figure S2**, cells infected with HCMV mutants lacking gp34 and/or gp68 did not show higher levels of opsonizing antigens on the plasma membrane. To compare the relative impact of gp68 versus gp34 on FcγRIII activation with a higher degree of accuracy, i.e. in the context of an identical HCMV genome possessing a preserved UL/*b′* gene region, another set of targeted vFcγR gene deletions was constructed based on the AD169varL derived BACmid pAD169 which carries unlike pHB5 only a single copy of *TRL* genes including *TRL11* [33]. As demonstrated in **Figure 2D**, targeted deletion of UL119-118/gp68 and TRL11/gp34 reproduced the increased activation of BW:FcγRIIIA-ζ responder cells, while combined deletion of both vFcγRs only marginally enhanced the response further. Next we determined if gp34 and gp68 could affect further activating host FcγRs and performed co-cultivation assays with MCR-5 cells infected with the same panels of HCMV mutants after opsonization with Cytotect and incubated with BW:FcγRIIA-ζ and BW:FcγRI-ζ reporter cells **(** **Figure 2C–D** **)**. While deletion of both HCMV Fcγ-binding proteins resulted in significantly enhanced responses by both FcγRIIA and FcγRI, the isolated removal of gp68 resulted in a slightly more drastic phenotype with regard to BW:FcγRIIA-ζ activation **(** **Figure 2D** **)**. Notably, combined removal of gp34 and gp68 led to a Δgp34-like phenotype, contrasting to the additive effect seen with FcγRIII at low IgG concentrations. In conclusion, the data suggested that both of the HCMV-encoded FcγRs might have developed the ability to interfere with the activation of FcγRIII, FcγRIIA and FcγRI, while HSV gE blocks FcγRIII and FcγRIIA but fails to inhibit FcγRI activation.

### *UL118-119* and *RL11* gene reversion restore resistance to FcγR activation by immune IgG

To exclude the possibility that second site mutations which occurred during the BACmid mutagenesis procedure are responsible for the observed loss of HCMV-mediated inhibition of host FcγR activation by immune IgG, an entirely independent panel of virus deletion mutants and the appropriate rescued versions were generated. The mutants were constructed using the HCMV TB40/E-derived BACmid [34] taking advantage of i) a single gene copy of *RL11* coding for gp34, ii) a complete HCMV UL*b′* gene region lacking in HCMV HB5 but present in HCMV clinical isolates and iii) a technically more feasible re-insertion strategy of the vFcγR coding genes. MRC-5 fibroblasts were left uninfected or infected with the HCMV TB40/E wt expressing gp68 and gp34, or with gp68 and gp34 single gene deletion mutants, resp., or independent single gene revertant mutants expressing gp68 or gp34. Using BW:FcγRIIIA-ζ responder cells and graded concentrations of HCMV immune IVIG, the gp34 and gp68 TB40/E deficient mutants elicited a stronger FcγR-ζ activation response than the TB40/E wt **(Figure S3A)**, while the density of opsonizing cell surface antigens was not altered **(Figure S3B)**. The finding that three independent virus mutants lacking Fc binding proteins show congruent phenotypes makes unintended second site mutations as cause for the effect highly unlikely. Nevertheless, revertant viruses were assessed. As expected, both of the revertant viruses exhibited a wt-like phenotype **(Figure S3A)**. In comparison to HCMV HB5, HCMV TB40/E shows a more protracted replication kinetic. Consistently, we observed more efficient IgG-dependent activation of FcγRIIIA-ζ at 96 hpi compared with 72 hpi. Therefore, HCMV TB40/E-based assays were performed 96 h post infection. The HCMV TB40/E results confirmed that both HCMV-encoded FcγRs inhibit the activation of FcγRIIIA and that their reinsertion into the virus genome reestablishes the vFcγR inhibition phenotype.

### Inhibition of IgG1 (trastuzumab) mediated activation of FcγRs

**Figure 3:**
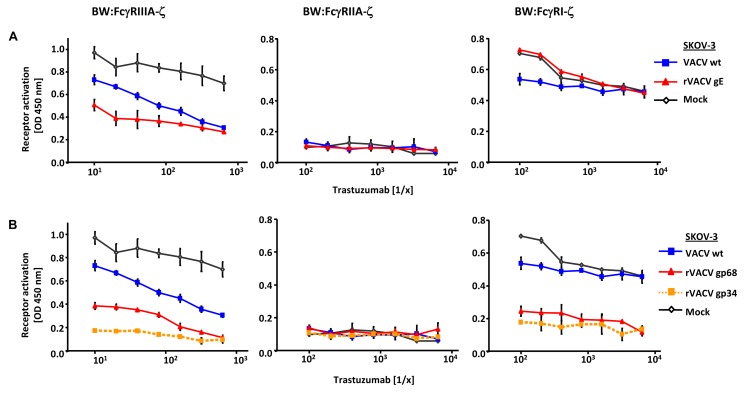
Ectopic expression of HSV-1 gE, HCMV gp68 and HCMV gp34 inhibit IgG1 (trastuzumab) mediated activation of FcγRs. (**A**) SKOV-3 cells were infected for 24 h with 2 PFU/cell VACV wt and rVACV expressing gE or (**B**) rVACV expressing gp68 or gp34 before opsonized with trastuzumab at different concentrations for 30 min. After removing of unbound antibodies by repeated washing with D-MEM 10% (vol/vol) FCS, 1×10^5^ BW:FcγR-ζ transfectants per well were added and co-cultivated overnight. BW:FcγR-ζ activation was determined by measuring mIL-2 by ELISA. Three independent replicates were measured, means with standard deviations (error bars) are shown for 3 independent experiments. Significance of results (Student's t-test) are presented in Table S1 as *: p<0.05 **: p<0.01 ***: p<0.001.

To test if gp34 and gp68 suffice to impair IgG-dependent activation of FcγRs, two factors of our experimental approach were modified: (i) gp34 and gp68 were expressed outside the context of HCMV infection by recombinant vaccinia viruses, and (ii) instead of polyclonal HCMV IVIG, a well-defined humanized therapeutic monoclonal IgG1 antibody (trastuzumab) was used as an activator of host FcγRs upon binding to its antigen HER2. rVACV expressing HSV gE-infected HER2 antigen positive SKOV-3 tumor cells were opsonized with graded concentrations of trastuzumab recognizing HER2 and compared with wt-VACV as well as mock-infected cells. The opsonized target cells were co-cultured with the panel of FcγR reporter cells **(** **Figure 3A** **)**. Opsonized VACV-infected cells exhibited a reduced capacity to trigger FcγRIIIA in comparison to mock cells, most likely due to the protein host shut-off function of VACV. Importantly, trastuzumab-mediated FcγRIIIA triggering was further impaired by rVACV gE, providing proof of principle that ectopically expressed gE suffices to interfere with IgG1-dependent FcγRIII activation. In contrast to FcγRIII, trastuzumab reproducibly failed to induce FcγRII responses **(** **Figure 3A** **)**. When trastuzumab-opsonized cells were probed with FcγRI transfectants, the presence of gE did not attenuate but rather enhanced the response **(** **Figure 3A** **)**, confirming the unexpected phenotype in the HSV-infected cell setting observed before **(** **Figure 2A** **)**. Next, rVACVs were used to express gp34 and gp68 ectopically in HER2 positive SKOV-3 targets which were opsonized with different concentrations of trastuzumab before co-culture with the same panel of responder cells as already described **(** **Figure 3B** **)**. Both gp34 as well as gp68 significantly reduced activation of FcγRIII and FcγRI, albeit in this setting gp34 seemed slightly more potent than gp68. In summary, deploying a gain-of-function approach and using a monoclonal human IgG1, the results verified that both HCMV FcγRs are sufficient to prevent the activation of FcγRI and FcγRIII.

### Interference with host FcγRIIA activation by ectopic expression of herpesviral FcγRs

Trastuzumab is not capable to activate FcγRIIA (see above, **Figure 3A**–**B**). Nevertheless, we wished to assess the effect of ectopically expressed vFcγRs on FcγRIIA activation. Therefore, in a further approach CD20 transfected 293T cells [35] were infected with rVACV expressing gE, gp68 or gp34 before opsonized with rituximab another well-defined humanized therapeutic monoclonal IgG1 antibody **(Figure S4A and S4B)**. All vFcγRs inhibited FcγRIIA activation verifying that ectopic expression of the viral Fcγ binding proteins gE, gp34 and gp68 hinder the activation of the host FcγRIIA in a gain-of-function approach.

### FcγRIII inhibition by gp34 and gp68 across IgG subclasses

**Figure 4:**
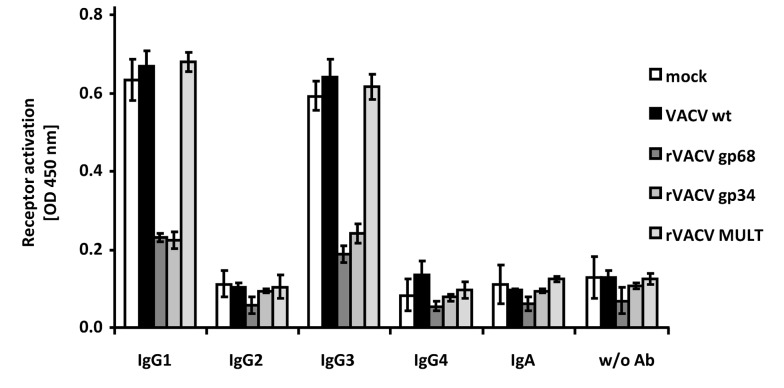
HCMV gp68 and gp34 inhibit FcγRIII activation by rituximab antibody isotypes. CD20 transfected 293T cells were infected for 16/cell of VACV wt or rVACV expressing gp68, gp34 or MULT-1 as a control. After opsonization with 1 µg/ml of each antibody isotype for 30 min. and removing of unbound antibody by washing, cells were co-cultivated with 1×10^5^ BW:FcγRIIIA-ζ reporter cells per well for 16 h before supernatants were collected and mIL-2 was determined by ELISA. Each value represents three replicates; means with standard deviations (error bars) are shown for 2 independent experiments. Significance of results (Student's t-test) are presented in Table S1 as *: p<0.05 **: p<0.01 ***: p<0.001.

Humans respond to HCMV infection with the production of IgG1 which is the immunodominant subclass, followed by IgG3, while HCMV-immune IgG2 and IgG4 is detected only at very low levels if produced at all [36], [37]. In contrast to HSV-1 gE, HCMV gp68 and gp34 bind monomeric IgG of all human subclasses, i.e. IgG1, IgG2, IgG3, and IgG4 [22], whereas gE does not bind IgG3 [38], [39]. To assess whether HCMV gp68 and gp34 can inhibit FcγRIIIA/CD16 activation through immune complexes formed by different IgG isotypes, we took advantage of a panel of rituximab-derived isotypic IgG antibodies. CD20 transfected 293T target cells [35] were infected with VACV wt or rVACV expressing gp68, gp34 or MULT-1 as a negative control and opsonized with anti-hCD20 IgG isotypes including an IgA constant region fused to the variable region of rituximab as an antibody control. CD20 expression revealed very similar levels of antigen expression on the cell surface of VACV target cells (data not shown). While opsonized IgG1 and IgG3 isotypes efficiently activated FcγRIIIA, very little to no activation was observed with IgG2, IgG4 and IgA, confirming previous data [28]. Both gp34 as well as gp68 strongly reduced activation of FcγRIIIA by IgG1 and IgG3 **(** **Figure 4** **)**. The data documented the inhibitory potency of both HCMV FcγRs against IgG1 and IgG3-formed immune complexes and confirmed the functional distinction of gp34 and gp68 against HSV gE.

### The herpesviral FcγRs inhibit antibody dependent NK cell degranulation

**Figure 5:**
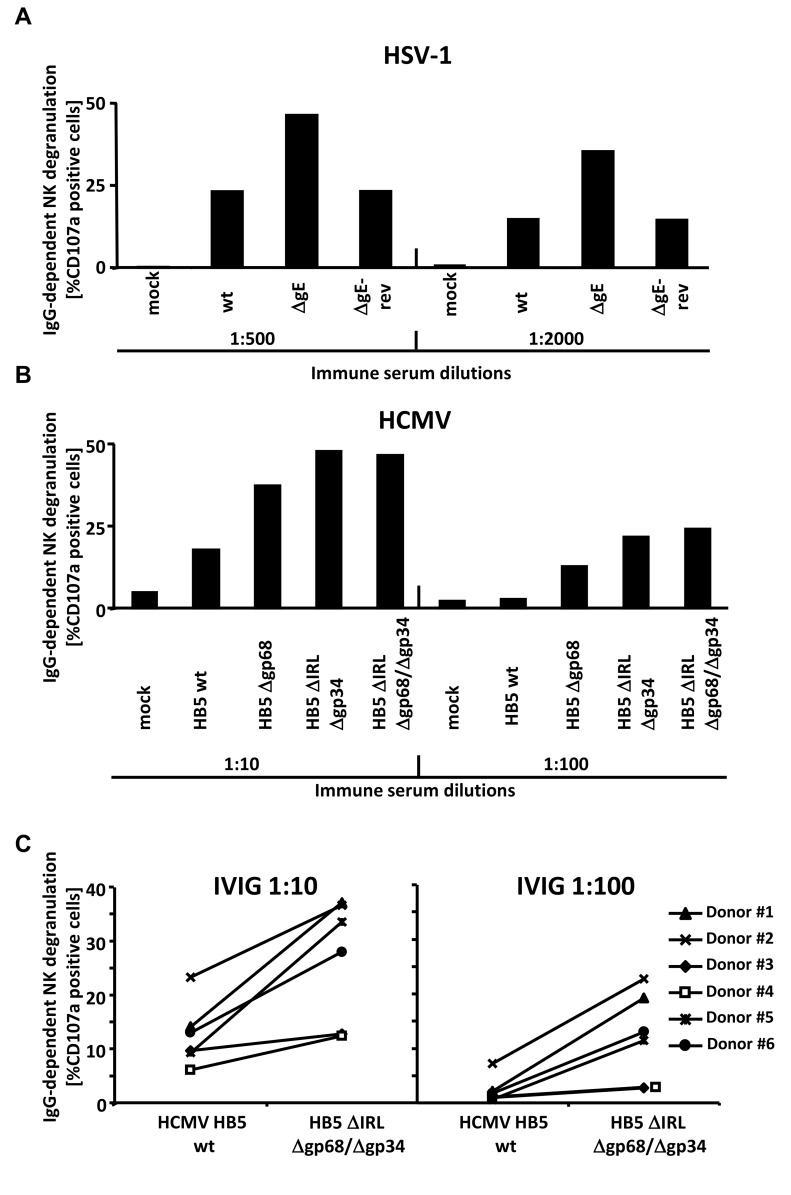
The presence of the vFcγRs on the surface of infected cells inhibits antibody dependent NK cell degranulation. (**A**) MRC-5 fibroblasts were infected with HSV-1F wt, ΔgE and ΔgE revertant for 24 h before cells were opsonized with HSV IgG positive and negative human sera. After 30 min of incubation, unbound antibodies were washed away and NK cells at an E∶T ratio of 10∶1 were added. After 4 h, CD107a surface expression on the NK cells was measured in FACS. Percentage of IgG-specific degranulation CD107a-positive cells = (% of CD107a-positive cells with the immune antibody opsonization - % of CD107a-positive cells with non-immune antibody). (**B**) MRC-5 fibroblasts were infected with HCMV HB5 wt, HB5Δgp68, HB5ΔIRLΔgp34 and HB5ΔIRLΔgp68/Δgp34 for 72 h before cells were opsonized with HCMV IgG positive and negative human sera. After 30 min of incubation, unbound antibodies were washed away and NK cells at an E∶T ratio of 10∶1 were added. After 4 h, CD107a surface expression on the NK cells was measured in FACS. Percentage of IgG-specific degranulation CD107a-positive cells = (% of CD107a-positive cells with the immune antibody opsonization - % of CD107a-positive cells with non-immune antibody) (**C**) MRC-5 fibroblasts were infected with HCMV HB5 wt and HB5ΔIRLΔgp68/Δgp34 for 72 h before opsonized with Cytotect or human HCMV-IgG negative sera at 1∶10 and 1∶100 dilutions. After 30 min of incubation, unbound antibodies were washed away and NK cells from six different donors at an E∶T ratio of 10∶1 were added. After 4 h, CD107a surface expression on the NK cells against HCMV-infected cells was measured. Percentage of IgG-specific degranulation CD107a-positive cells = (% of CD107a positive cells with the immune antibody opsonization - % of CD107a positive cells with non-immune antibody). Single measurements of CD107a cells are shown. One of three (A, B) or two (C) representative experiments is shown.

CD16/FcγRIII is an essential IgG receptor for activation of NK cells mediating ADCC responses [40], [41] but also found on human γδ T cells induced by HCMV infection [42]. The data obtained with the FcγRIII-ζ reporter cells strongly suggested that gp34, gp68 as well as HSV-1 gE operate as inhibitors of FcγRIII/CD16+ NK cells since BW:FcγRIII-ζ responses showed an excellent match with CD107a mobilization of primary human NK cells upon CD16/FcγRIII cross-linking [28]. Therefore, we tested the activation of primary human NK cells by fibroblasts infected with HSV-1, HCMV and mutants devoid of viral Fcγ binding proteins, respectively, in the presence of virus-immune opsonizing IgG in a CD107a degranulation assay [43]. The sources of the opsonizing IgG were sera donated by HSV/HCMV-seropositive donors **(** **Figure 5A** **and** **Figure 5B** **, resp.)** or Cytotect **(** **Figure 5C** **)**. rhIL-2 overnight preactivated NK cells from HSV/HCMV sero-negative donors were enriched by negative selection and analyzed after 4 hours of co-incubation with infected cells opsonized with graded concentrations of immune IgG. HCMV encodes numerous inhibitors of NK cell activation [44], [45]. To focus on IgG-dependent NK cell activation, NK activation was calculated and depicted as percentage of IgG-specific CD107a mobilization (i.e. percentage of CD107a-positive cells obtained with the immune antibody opsonizing target cells minus the percentage of CD107a-positive cells obtained with non-immune antibody treated target cells). A higher ratio of IgG-dependent CD107a positive cells in the case of HSV-1 ΔgE-infected cells compared with wt HSV-1 infected cells was observed **(** **Figure 5A** **)**. Likewise, the HB5Δgp68, HB5ΔIRLΔgp34 and HB5ΔIRLΔgp68/Δgp34 HCMV mutants yielded clearly increased IgG-dependent CD107a mobilization as HCMV HB5 **(** **Figure 5B** **)**. As observed with BW:FcγR-ζ responder cells, gp34 and gp68 inhibited FcγRIIIA NK activation independently, but no additive effects were noted upon deletion of both vFcγRs. To exclude donor-specific effects, NK cells from six different donors were analyzed in degranulation assays comparing HCMV HB5 wt with HB5ΔIRLΔgp68/Δgp34 -infected targets opsonized with Cytotect as a source of immune IgG and non-immune sera as a negative control. All donors showed a higher percentage of IgG-dependent CD107a positive cells in the case of HB5ΔIRLΔgp68/Δgp34 -infected cells **(** **Figure 5C** **)**. Taken together, these data demonstrated that vFcγR gE, gp34 and gp68 on the surface of infected cells mediate inhibition of IgG-dependent NK cell degranulation.

### HCMV FcγRs form ternary complexes with antigen and IgG on the cell surface compatible with antibody bipolar bridging

**Figure 6:**
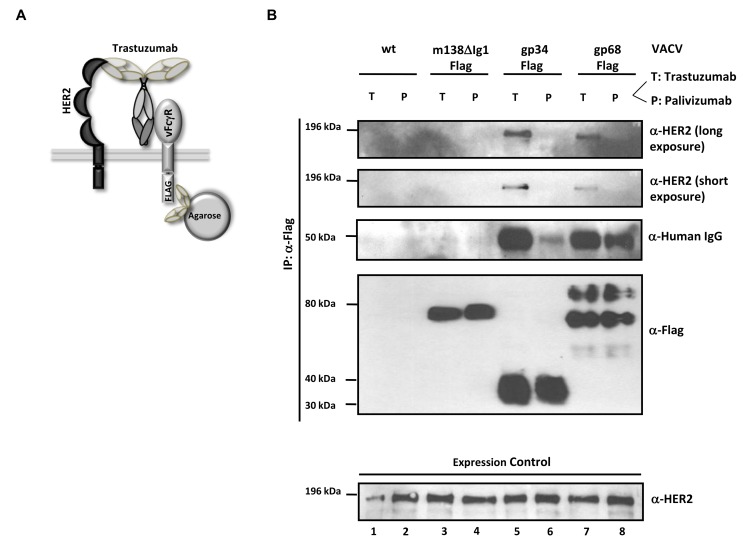
The HCMV vFcγRs gp68 and gp34 bind antigen-IgG complexes. (**A**) Schematic representation of the ‘antibody bipolar bridging’ model. (**B**) Lysates of SKOV-3 cells containing the heterocomplex of vFcγR-FLAG, antibody and antigen are immunoprecipitated using an anti-FLAG agarose. SKOV-3 cells expressing HER2 antigen on the surface were infected with rVACV expressing the vFcγRs before opsonized with the trastuzumab antibody (bipolar bridging-antibody) (T) or an isotype control IgG1 antibody, palivizumab (P). Lysates were prepared after incubation of infected cells with antibody. An anti-Flag agarose IP was performed and retrieved antigens were detected in western blot with anti-ErbB2-specific mAb recognizing human HER2, anti-human IgG, and anti-Flag (M2, Sigma-Aldrich) detecting the Flag-tagged vFcγRs. Equal expression of HER2 in cell lysates was verified by western blot analysis with an anti-ErbB2-specific rabbit mAb which detects human HER2 (bottom).

HCMV gp68 was found to bind the Fc C_H_2-C_H_3 interface of monomeric IgG at nanomolar affinity [24]. To get insight into the intermolecular interactions underlying the inhibitory function of gp34 and gp68 when blocking antigen-antibody complexes, we tested the occurrence of a physical complex on the surface of cells consisting of the target antigen, bound IgG and each of the HCMV FcγRs. We took advantage of immune complexes (composed of trastuzumab and its antigen HER2) which were shown to be sensitive to the blockade through gp34 and gp68 when activating FcγRIII and FcγRI **(** **Figure 3B** **)**. HER2-expressing SKOV-3 cells were infected with rVACV expressing Flag-tagged gp34, gp68 or a control protein, ΔIg1-m138, a non-functional MCMV *m138*/fcr-1 truncation mutant [46] and opsonized with trastuzumab (‘T’) or with an IgG1 isotype control antibody, palivizumab (‘P’) (see sketch in **Figure 6A**). VACV-infected cells were thoroughly washed to remove unbound antibodies and subsequently lysed. To exclude Fcγ-mediated binding of vFcγRs through anti-Flag antibodies, vFcγR proteins were immunoprecipitated using α-Flag F(ab)_2_-coupled agarose beads. The precipitated proteins were separated by SDS-PAGE and analyzed by immunoblotting using an HER2 specific antibody **(** **Figure 6B** **)**. An anti-human IgG-specific antibody was used to detect the co-precipitated antibody. An immuno blot confirmed expression and immunoprecipitation of the vFcγRs. Retrieval of palivizumab by gp34 and gp68 was weaker than retrieval of trastuzumab. This difference could be explained by the fact that trastuzumab could be retained by the cells via vFcγRs and via HER2, while palivizumab could only be retained by vFcγRs. Subsequently, gp34 and gp68 retrieved trastuzumab antibodies bound to HER2 during lysis and precipitation. Nevertheless, co-precipitation of human HER2 molecules occurred only in the presence of specific antibody trastuzumab and the HCMV FcγRs but not in the negative control ΔIg1-m138 **(** **Figure 6B** **, lanes 5, 7 and 3, respectively)**. Binding of human IgG antibodies, trastuzumab and the isotype control antibody palivizumab, was observed to both vFcγRs **(** **Figure 6B** **, lanes 5, 6, 7 and 8)**. No binding of trastuzumab or palivizumab was detectable to the ΔIg1-m138- Flag protein **(** **Figure 6B** **, lanes 3 and 4)**. Taken together, the ability of cell surface resident vFcγRs gp34 and gp68 to bind to IgG immune complexes was demonstrated. This finding is compatible with the model of “antibody bipolar bridging” described for the HSV-1 FcγR gE [47]–[49]. According to this concept, epitope-bound IgG on the surface of a virus-infected cell is simultaneously sequestered by the gE:gI complex via its Fc domain, thus preventing the activation of immune effector molecules via host FcγRs.

### Soluble ectodomains of HCMV vFcγRs inhibit IgG-dependent host FcγRs activation

**Figure 7:**
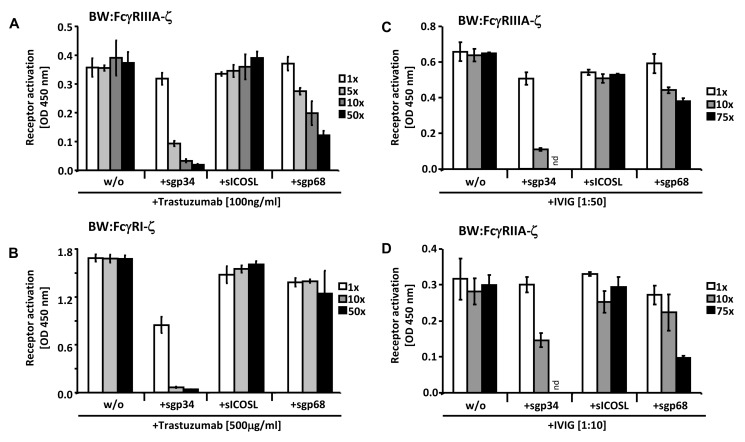
Soluble ectodomains of HCMV vFcγRs interfere with FcγR activation. (**A**) SKOV-3 cells were opsonized with 100 ng/ml trastuzumab for 30 minutes and washed three times with D-MEM 10% FCS (vol/vol) before soluble proteins were added in graded concentrations concomitantly with BW:FcγRIIIA-ζ transfectants. mIL-2 was determined in supernatants (which were harvested after 16 h of co-cultivation of responder cells with target cells) by ELISA. (**B**) As in (A) but SKOV-3 cells were opsonized with 500 µg/ml trastuzumab for 30 minutes and washed three times with D-MEM 10% FCS (vol/vol) before soluble proteins were added in graded concentrations concomitantly with BW:FcγRI-ζ cells. (**C**) MRC-5 cells were infected with HCMV HB5ΔIRLΔgp68/Δgp34 (2 PFU/cell) for 72 h before soluble proteins were added in graded concentrations concomitantly with BW:FcγRIIIA-ζ responder cells. MRC-5 fibroblasts were opsonized with 1∶50 diluted Cytotect for 30 min. After removing of unbound antibodies by washing, soluble proteins and BW:FcγR-ζ transfectants were added and co-cultivated overnight. (**D**) as in (C), but HCMV HB5ΔIRLΔgp68/Δgp34 infected target cells were opsonized with 1∶10 diluted Cytotect and BW:FcγRIIA-ζ cells were used as responders. n = 3 replicates, means with standard deviations (error bars) are shown for 2 independent experiments. Significance of results (Student's t-test) are presented in Table S1 as *: p<0.05 **: p<0.01 ***: p<0.001.

Soluble truncation versions of HSV-1 gE and HCMV gp68 were instrumental to unravel structural requirements and stoichiometry of herpesviral FcγRs forming complexes with Fcγ [24], [49], [50]. To test whether membrane insertion of gp34 and gp68 is required to interfere with the activation of host FcγRs, recombinant C-terminally truncated ectodomains of HCMV FcγRs were generated and purified from supernatants of transfected human 293 cells. To evaluate if soluble gp34 (sgp34) and soluble gp68 (sgp68) are sufficient to block triggering of host Fcγ receptors, HER2-positive cells were opsonized with trastuzumab and different amounts of recombinant soluble vFcγRs were concomitantly added to BW:FcγRIIIA-ζ cells **(** **Figure 7A** **)** or BW:FcγRI-ζ cells **(** **Figure 7B** **)**. Soluble ICOS ligand (sICOSL) served as a negative control protein. Both sgp34 and sgp68 were able to inhibit activation of the reporter cells expressing FcγRIIIA, although clear differences in concentration dependency between soluble vFcγRs were observed. In contrast to full-length HCMV FcγRs, sgp34 was more potent against FcγRIIIA compared to sgp68, since trace amounts of sgp34 hardly detectable in western blot **(Figure S5)** were sufficient for significant inhibition. In the case of FcγRI, sgp68 was not significantly reducing its activation by trastuzumab, suggesting the specific requirement of the gp68 transmembrane domain for effective inhibition of FcγRI/CD64.

To extend the data to HCMV infection and to test BW:FcγRIIA-ζ cells, a polyclonal antibody preparation (IVIG, Cytotect) was used to opsonize MRC-5 fibroblasts infected with the HCMV HB5ΔIRLΔgp68/Δgp34 mutant lacking both gp68 and gp34. Using BW:FcγRIIIA-ζ reporter cells, both of the soluble HCMV vFcγRs prevented activation when compared with treatment of cells with the sICOSL control **(** **Figure 7C** **)**. Moreover, this approach allowed to test activation of BW:FcγRIIA-ζ cells which did not respond to trastuzumab (see **Figure 3**). As depicted in **Figure 7D**, FcγRII responses were also sensitive to sgp34 and, to a lesser extent, sgp68. These results provide proof of principle that soluble HCMV FcγRs retain FcγR blocking abilities, and that sgp34 is particularly efficient.

### Soluble ectodomains of HCMV vFcγRs inhibit IgG-dependent NK cell degranulation

**Figure 8:**
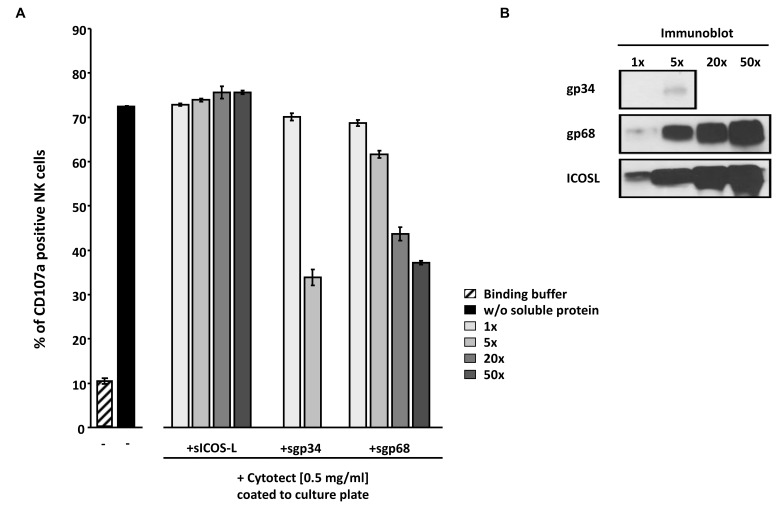
Soluble ectodomains of HCMV vFcγR interfere with antibody dependent NK cell degranulation. (**A**) Cytotect was coated to a plate in binding buffer (0.1 M Na_2_HPO_4_ pH 9.0) at a concentration of 0.5 mg/ml and incubated for 2.5 hours at 37°C. After blocking for 30 minutes and washing unbound antibodies, soluble proteins, rIL-2 pre-activated primary NK cells and α-CD107a-PECy5 antibody were added and incubated for 4 hours at 37°C. Duplicates were measured for CD107a surface expression after dead cell exclusion with DAPI staining in a FACS Canto II. Means are shown with standard deviations (error bars). Significance of results (Student's t-test) are presented in Table S1 as *: p<0.05 **: p<0.01 ***: p<0.001. (**B**) To compare the amounts of soluble proteins used in (A), SDS-PAGE and anti-V5 immunobotting was performed.

In an attempt to extend the previously made observation of sgp34 and sgp68-mediated inhibition of IgG-triggered FcγRIII/CD16+ BW5147 responder cells to primary human NK cells, purified IVIG Cytotect was coated directly to a plate serving as a source of ‘immune-complexed’ IgG. After blocking with D-MEM 10% FCS (vol/vol), soluble proteins, IL-2 pre-activated primary NK cells and α-CD107a-PECy5 antibody were added. Soluble ICOS ligand (sICOSL) served as a negative control protein. In the absence of coated IgG, only 10% of NK cells responded with CD107a mobilization, while in the presence of coated IgG more than 70% of NK cells translocated CD107a to the cell surface **(** **Figure 8A** **)**, confirming the IgG-dependency of the elicited NK cell response. Importantly, both sgp34 and sgp68 were able to inhibit IgG-dependent NK degranulation, although a clear difference in the concentration dependency between soluble vFcγRs was observed. Consistent with the results received with BW:FcγRIIIA-ζ reporter cells **(** **Figure 7C** **)**, sgp34 was more potent against IgG-dependent NK activation compared to sgp68, since trace amounts of sgp34 **(** **Figure 8B** **)** were sufficient to significantly interfere with degranulation of NK cells. Using a gain of function approach the data confirmed the inhibitory capacity of gp34 and gp68 to attenuate IgG-mediated NK cell activation.

## Discussion

Here we identified various members of the human FcγR family, i.e. FcγRI/CD64, FcγRII/CD32A and FcγRIII/CD16A, to be targeted by the HCMV FcγRs gp34 and gp68 which act as antagonists of ligand induced FcγR responses. This ability enables HCMV to evade from IgG effector responses and should have direct proviral effects in scenarios of post-acute and recurrent infection when glycoprotein-specific IgG antibodies are synthesized [51]. Several independent experimental approaches support this conclusion: i) HCMV HB5-derived mutants with deletions of the gp34 and gp68 coding genes, *TRL11/IRL11* and *UL118-119*, respectively, showed significantly increased activation of host FcγRs upon opsonization of infected cells with polyclonal HCMV immune IgG using different types of responder cells (i.e. BW5147 transfectants expressing FcγR-ζ chain chimeras and primary human NK cells); ii) this HCMV phenotype was reproduced with targeted *RL11* and *UL118-119* mutants of the AD169varL and TB40/E strain and iii) fully reversed by retransfer of the responsible genes into the *RL11-* and *UL118-119*-deficient TB40/E genomes; iv) a gain-of-function approach based on ectopic VACV-based expression of gp34 and gp68 which allowed functional analysis of well characterized therapeutic human monoclonal antibodies and different IgG isotypes thereof and v) functional testing of recombinant soluble ectodomains of both HCMV inhibitors using BW5147:FcγR reporter cells as well as primary human NK cells. Importantly, the inhibitory effect of gp34 and gp68 was demonstrated at physiological concentrations of polyclonal HCMV immune IgG, i.e. within an extended concentration range of human serum and ten to fifty fold lower.

The quite complex and overlapping expression patterns of host Fc-IgG receptors on a multitude of diverse human immune cell (sub-)populations [17] have obstructed a systematic functional analysis of individual host FcγRs which differ with respect to molecular and functional features including the composition of their ectodomain, intracellular signaling and IgG subclass preferences. Only a recently developed methodologically broadly tested and proven reporter cell assay [28] enabled a comprehensive and quantitative functional assessment of potential viral antagonists and their relative effectiveness against distinct host FcγRs. The new methodology was complemented and validated by immunological as well as biochemical assays, i.e. the use of primary human NK cells as natural responder cells and immunoprecipitation studies, the results of which accord very well with the findings made with the BW5147:FcγR-ζ test system. Last but not least, the well-known viral FcγR inhibitor, HSV gE, was included as an internal control. Previous publications reported inhibition of ADCC, virion neutralization and complement mediated virolysis by HSV gE [47], [52], [53]. Since the blockade of ADCC by gE was not yet attributed to a specific host FcγR [30], [47], [54], analysis of the relative impact of HSV gE on distinct host FcγRs represents a novel aspect of our study. On the basis of the test performance of HSV gE, both gp34 and gp68 demonstrated an at least equivalent if not superior efficacy to block FcγRIII and FcγRIIA mediated responses. Surprisingly and contrasting with both HCMV vFcγRs, gE enhanced rather than attenuated FcγRI activation. This observation warrants further studies how HSV-infected cells affect FcγRI bearing immune cells like monocytes, macrophages, DCs and neutrophils in the presence of HSV-immune IgG.

### Structural requirements for FcγR inhibition

The inhibition mechanism of IgG-mediated effector functions by gE has been suggested to involve ‘antibody bipolar bridging’ [47]. Pioneering studies of the Bjorkman laboratory demonstrated that the architecture of the gE/gI-IgG complex allows antibody bipolar bridging [49], whereby the gE binding site for Fcγ does not directly overlap with the binding sites to the host FcγRs or the C1q component of complement, which both bind to the upper hinge region of IgG or near the C_H_2 domain [55], [56]. Therefore, the structure of the gE/gI-Fc complex does not directly explain how gE binding to the Fc region of IgG leads to evasion from FcγR- and complement-mediated immune responses. Our biochemical data reveal formation of ternary heterocomplexes composed of antigen, IgG and gp34/gp68, i.e. a molecular configuration compatible with the minimal requirements of the concept of ‘bipolar bridging’ [47], [49]. The observation that soluble gp34 and gp68 remain potent inhibitors of FcγR activation demonstrates that the functional inactivation of the host FcγR on the responder cell does not require fixation of the opsonized IgG to the plasma membrane as insinuated by the classical concept of bipolar bridging. Although there is no crystal structure available for any HCMV vFcγR, detailed biochemical evidence was generated of how HCMV FcγRs recognize Fcγ, particularly for gp68. The gp68 Fcγ binding site was mapped to the C_H_2-C_H_3 interface region of Fcγ [24] which is remote from the FcγRII/III contact site, that involves the hinge between the Fcγ and Fab domains including the upper portion of the C_H_2 domain [55], [57]. Specifically, gp68 binding to Fcγ is affected by mutations at the C_H_2-C_H_3 domain interface of IgG, mapping its binding site to determinants situated nearby but not identical with those that are recognized by gE [24]. We observed robust functional differences between HSV-1 gE and HCMV gp68 in their manipulation of FcγRI, further substantiating the mechanistic differences between these viral inhibitors regarding their interaction mode with Fcγ. While the binding of IgG by gE must induce conformational changes of the antibody that result in an enhancement of FcγRI activation, gp68 binding to IgG induces the opposite effect. Since both gE and gp68 had concordant inhibitory effects on FcγRIII, our data further imply that FcγRI and FcγRIII must bind IgG in a differential fashion. Importantly, the length and flexibility of the hinge region varies considerably among the IgG subclasses, and IgG3 differs from the other subclasses by its unique extended hinge region which is approx. four times as long as the IgG1 hinge, leading to the most hinge-mediated flexibility among human IgG subclasses [58]. Notably, we demonstrate efficient FcγRIII blockade by IgG3-shaped immune complexes through gp68 and gp34 **(** **Figure 4** **)** which is not possible by HSV gE [36], [37]. Thus, by analogy with HSV gE, HCMV gp34 and gp68 represent promising and unique tools to further probe into the diverse structural requirements of FcγRI/II/III activation by immune complexes constituted by all IgG subclasses.

### Functional redundancies of gp34 and gp68?

The presence of independent but redundant immunoevasins jointly targeting one particular immune control mechanism is a typical feature of cytomegaloviruses highlighting the antiviral power of the targeted immune component [6], [59]–[62]. At first glance, the HCMV FcγR antagonists gp34 and gp68 exhibit a surprisingly similar effect on the whole range of activating host FcγRs, despite their simultaneous synthesis during the early and late phase of HCMV replication [22]. As a consequence, removal of both inhibitors from the surface of HCMV infected cells did not reveal additive or even synergistic effects compatible with the notion that the two factors do not act in an obvious cooperative manner. This finding cannot be attributed to differences in the density of plasma membrane resident HCMV antigens between the HCMV gene deletion mutants compared in our study (see **Figure S2 and S3**). However, both of the antagonists could themselves represent antigens that are recognized by the F(ab) part of immune IgG, which could either directly activate host FcγRs or block Fcγ-mediated bridging of opsonizing IgG (as a counter defense of humoral immunity against vFcγRs) and thus indirectly enhance host FcγR triggering. Moreover, both of the HCMV FcγRs may fulfill further proviral but Fcγ-independent functions which exert separate pressures to adapt. This is exemplified by the MCMV *m138*/fcr-1 molecule which down-regulates the NKG2D ligands MULT-1, H60, RAE-ε [46], [63] as well as the B7-1 molecule CD80 [64] beyond its Fcγ binding activity. In addition, besides gp34 and gp68 additional HCMV FcγRs become expressed on infected cells (Mercé-Maldonado and Hengel, in preparation), one of which is encoded by *RL13* [65]. Thus gp34 and gp68 may be part of a much more complex network of co-expressed HCMV FcγRs jointly combating their host opponents, and removal of one player could confound their interplay and nested hierarchies. Next, drastic quantitative and qualitative differences in the potency of gp34 vs. gp68 became apparent when soluble molecules were compared. Thus it is tempting to speculate that shedding of vFcγRs may be part of the molecular blueprint of particular vFcγRs.

### Which HCMV antigens elicit ADCC responses?

While there is extensive knowledge on antigens and processed epitopes which rule anti-HCMV T cell responses [66], viral antigens that are targets of ADCC dependent cellular immunity remain poorly defined. Our finding that late but not early antigens dominate the FcγRIII/CD16 activating IgG response **(** **Figure 1B** **)** appears a particular characteristic of HCMV when compared with HSV and could point to structural glycoproteins known to become exposed on the cell surface as the HCMV replication cycle progresses, e.g. gB [67], gH [68] and UL128 [69]. Guided by human antibodies with defined specificity, our BW5147-based FcγR-ζ assay system could be instrumental to identify the relevant HCMV antigens and epitopes. In many tissues and organ compartments, including blood, HCMV is spreading intracellularly (e.g. via infected endothelial cells and leukocytes) rather than as free virions [70]. Therefore, ADCC-inducing IgG is plausible to represent a primary effective component of humoral immunity, which becomes only secondary attenuated by gp34 and gp68. Both immunoevasins could thus contribute to the relatively poor therapeutic efficacy of HCMV-immune IgG observed in a variety of clinical settings [12]–[14]. Thus a better knowledge of the optimal HCMV IgG epitopes on the one hand, and an understanding of the action of viral FcγR antagonists on the other hand, could provide us with a basis for the targeted induction or even rational synthetic design of IgG molecules that allow an improved immunotherapy of HCMV diseases.

## Materials and Methods

### Cell lines, viruses and infection conditions

Human MRC-5 lung fibroblasts (ATCC CCL-171), African green monkey CV-1 (ATCC CCL-70), HEK293 (ATCC CRL-1573) and CD20 transfected 293T cells (a kind gift from Irvin S. Y. Chen, University of California) [35] cells were maintained in culture with D-MEM (Gibco), 10% (vol/vol) heat-inactivated FCS, Penicillin (100 U/ml), Streptomycin (100 µg/ml) and Glutamine (2 mM). Mouse BW5147 thymoma cells (obtained from ATCC, TIB-47), transfectants thereof [28] and SKOV-3 cells were maintained in RPMI 1640 medium with 10% (vol/vol) FBS, Penicillin, Streptomycin, Glutamine, and Sodium Pyruvate (1 mM). The following viruses were used: the bacterial artificial chromosome plasmid (BACmid)-derived human cytomegalovirus (HCMV) strain HB5 [71], the HB5-derived mutants lacking *UL118-120* (Δgp68) [22] or lacking *IRL/RL11/UL118* (Δgp68/Δgp34) [31], TB40/E BAC [34], herpes simplex virus 1 strain F (HSV-1), HSV-1 ΔgE and revertant thereof [72], VACV wt Western Reserve, recombinant VACVs expressing the vFcγRs [22], [73], a rVACV expressing MULT-1 [74] and a rVACV expressing a non-IgG binding truncation mutant of MCMV *m138* (m138ΔIg1Flag) [46]. The HB5-derived deletion mutant *TRL10-14/IRL* (Δgp34) was constructed following a previously published procedure [75]. The BAC plasmid pHB5-ΔIRL-frt (kindly provided by Eva Borst, Hannover, Germany), lacking the nucleotides 179150–192329 of the AD169-derived BAC pHB5 [71] was used to generate BAC plasmid pΔIRL/ΔTRL11 (lacking the viral FcγR gp34; Δgp34). For its construction, the primer pair AZ-TRL11-tet1 5′ACAGACGACGAAGAGGACGAGGACGACAACGTCTGATAAGGAAGGCGAGAACGTGTTTTGTCCAGTGAATTCGAGCTCGGTAC-3′ and AZ-TRL11-tet2 5′TGTATACGCCGTATGCCTGTACGTGAGATGGTGAGGTCTTCGGCAGGCGACACGCATCTTGACCATGATTACGCCAAGCTCC-3′ was used to amplify the tetracycline resistant gene (*Tet^R^*) from pCP16 for insertion into pΔIRL-frt. Recombinant TB40 HCMV were generated according to a previously published procedure [75] using BAC plasmid TB40/BAC4 [34]. For construction of the TB40 Δgp34 mutant, a PCR fragment was generated from the contiguous primers PL-delRL11-1 (5′-TCCCCGTTGATCGAACCGACGGGCACAGACGACGAAGAGGACGAGGACGACGACGTCTGACCAGTGAATTCGAGCTCGGTAC-3′) and PL-delRL11-2 (5′-CATGCATGTTATTTGCGTGTACGATGACTTGTTTCGCCGTCGATGTTGTGTACGCATCTTTTACTCCAAATCCCCGTCCACCCACCATGATTACGCCAAGCTCC-3′) using the plasmid pSLFRTKn [22] as template DNA. For construction of the TB40 Δgp68 mutant a PCR fragment was generated from the contiguous primers PL-delUL119-1 (5′-GGTCTCCTGCGGCCTGAGTCCCGAGATAAGCAGCTCTTGAGCAGTAGCGTTGTAGGAGAGCCAGTGAATTCGAGCTCGGTAC-3′) and PL-delUL119-2 (5′-AGGTGACGCGACCTCCTGCCACATATAGCTCGTCCACACGCCGTCTCGTCACACGGCAACGACCATGATTACGCCAAGCTCC-3′) using the plasmid pSLFRTKn [22] as template DNA. The PCR fragments containing a kanamycin resistance gene were inserted into TB40/BAC4 by homologous recombination in *E. coli*. The *Kn^r^* was excised from both BACs by flp-mediated recombination [75] generating the HCMV BACs TB40-Δgp34 and TB40-Δgp68. The revertant viruses were constructed from PCR-fragments containing the respective gene and flanking homologies subcloned into the *Bam*HI site of the shuttle vector pST76KSR. The following primer pairs were used: AZ-RL11rev-1: GtGGATCCGAGTGTTGAAGGGTAACGTGAGGGA and AZ-RL11rev-2 GCTCTAGAGCATGCAGATCTGTCTTGTAGCACGATGTGGTGGT for the gp34 revertant and AZ-UL118rev-1: GCTCTAGAGCATGCAGATCTACCACTGCTTGAAGTAGGGCACC and AZ-UL118rev-2: GtGGATCCGGTGGTATGAGCCTGAAGTGAGCAT for the gp68 revertant. The viral FcR genes from plasmids pST76KSR-RL11rev and pST76-KSR-UL119rev were inserted into TB40-Δgp34 and TB40-Δgp68, respectively, by two-step homologous recombination [76] resulting in the BACs TB40-gp34Rev and TB40-gp68Rev. Correct mutagenesis of all recombinant HCMV-BACs was confirmed by restriction analysis and sequencing of the respective genome region. Recombinant viruses TB40-Δgp34, TB40-Δgp68, TB40-gp34Rev and TB40-gp68Rev were reconstituted by transfection of MRC-5 using Superfect reagent (Qiagen, Germany) as described by Borst *et al.* [71]. The AD169varL deletion mutants were generated according to a previously published procedure [75] using the BACmid-cloned AD169varL genome pAD169 [33] as parental BACmid. Briefly, a PCR fragment was generated using primers listed in Table S2. The PCR fragment containing a kanamycin resistance gene was inserted into the parental BACmid by homologous recombination in *E. coli*. For the construction of mutants harboring deletions of two non-adjacent genes, the kanamycin cassette was removed by flp-mediated recombination before introducing the second deletion. Recombinant mutant viruses were reconstituted from BACmid DNA by Superfect (Qiagen, Germany) transfection into HCMV-permissive MRC-5 fibroblasts.

Infection of cells with HCMV and HSV was enhanced by centrifugation at 800 *g* for 30 min. If not stated otherwise, the cells were infected with 2–3 PFU/cell.

### Human immunoglobulin preparations, human serum pools and humanized antibodies

A clinically used IVIG preparation [11] Cytotect [32], [77] (batch no. A158024 and B797053, Biotest Pharma GmbH, Germany) containing ELISA reactive IgG specific for HCMV and HSV was used. For the FACS analysis of HCMV and HSV surface antigens, F(ab)_2_ fragments were generated from Cytotect with the Pierce F(ab)_2_ Micro Preparation Kit (Thermo Fisher Scientific Inc., Rockland, IL, USA) according to the manufacturer's instructions and controlled by Western Blot (data not shown). For the experiments with HCMV and HSV, a pool of two ELISA seronegative donors were used as a negative control. Trastuzumab was purchased from Genentech, Inc., USA and palivizumab from MedImmune, USA. The humanized anti-CD20 IgG1, IgG2, IgG3, IgG4 isotypes and IgA were purchased from InvivoGen, Toulouse, France. For the CD107a NK degranulation assay, an HCMV- and HSV-seropositive donor and a negative serum donor as sources of immune and non-immune IgG, respectively, were used. For proofing that the assay was antibody-antigen specific (Figure S1), a pool of 6 HCMV- and HSV-seropositive donors and a pool of 2 negative serum donors as sources of immune and non-immune IgG, respectively, were used. For serum preparation, blood was drawn from healthy volunteers after written informed consent.

### Ethics statement

The experiments were approved by the Ethics Committee of the University Hospital Düsseldorf (no. 3410) in accordance with the Declaration of Helsinki. For serum and NK cells preparation, blood was drawn from healthy volunteers after written informed consent.

### IgG dependent activation of the BW:FcγR-ζ transfectants

This assay was described elsewhere [28]. Briefly, in a standard assay, target cells were incubated with dilutions of human sera, IVIG, the anti-hCD20 IgG isotype collection or trastuzumab in D-MEM with 10% (vol/vol) FCS for 30 min at 37°C. Cells were washed before co-cultivation with BW:FcγR-ζ transfectants (ratio E∶T 20∶1) for 16 h at 37°C in a 5% CO_2_ atmosphere. Then mIL-2 secreted was measured by ELISA. When applied to VACV infected cells, a previous step of UV-inactivation at 4000 Jules/m^2^ and 2 steps of washing with PBS were performed before opsonization with Abs. When applied to the inhibition through soluble vFcγR, soluble proteins were added concomitantly with BW:FcγR-ζ transfectants. For herpesviral late antigens IgG-dependent activation of BW:FcγRIIIA cells, late phase gene expression was blocked by the use of phosphonoacetic acid (PAA) (250 µg/ml), which blocks viral genome replication and late gene expression. Afterwards, a co-cultivation assay was performed as described above.

### Expression and purification of soluble V5-His tagged HCMV vFcγR ectodomain proteins

The N-terminus of gp34 was amplified by PCR using the following primers 5′-GCTTAG*GGATCC*ATGCAGACCTACAGCACCCC-3′) [22] and 5′-TCTC*ACTAGT*GGACCACTGGCGTTTTAAATC-3′. Cloning of the N-terminus of gp68 was previously described [24]. The N-terminus of ICOSL (ICOS ligand) was amplified by PCR using the following primers 5′-GAGGTA*AGATCT*CGCACCATGCGGCTGGGC-3′ and 5′-CTCTC*ACTAGT*CGTGGCCGCGTTTTTC-3′. Sequencing of the coding sequences showed an amino acid exchange in ICOSL from V_128_ to I_128_ but with no detectable functional difference. Each PCR product was cloned in pGene/V5-His B vector (Invitrogen, USA) in frame with the V5-His epitope tag using the restriction sites *Bgl*II and *Spe*I (italics in primer sequences) and then subcloned in pIRES-EGFP vector (Clontech, USA). For enhanced expression of gp34V5-His and gp68V5-His, γ-Globin cloned from pSG5 vector (Stratagene, USA) was inserted into pIRES-EGFP between the CMV-IE promoter and the coding sequences. The plasmids were transfected in HEK293 cells using Superfect (Qiagen, Germany) and transfected cells were selected with 1.25 mg/ml of Geneticin (Sigma-Aldrich, Germany). After 4–5 days, supernatants were collected, volume reduced, diluted with PBS (1∶3), adjusted to a 10 mM Imidazole concentration and passed over a His-Trap FF crude column (GE Healthcare, USA). Proteins were eluted in Imidazole/Phosphate buffer (250 mM Imidazole, 20 mM sodium phosphate, 500 mM NaCl) and then dialyzed to PBS. Comparable protein amounts were adjusted based in Western blot analysis using α-V5 antibody (Invitrogen, USA).

### FACS analysis for vFcγR expression on infected cells

MRC-5 cells were infected with 2 PFU/cell of wt HCMV and HCMV vFcγR mutants during 72 h and with HSV-1 wt, ΔgE and ΔgE-revertant during 24 h. Cells were resuspended in PBS containing 2 mM EDTA, washed twice in PBS supplemented with 3% (vol/vol) FCS and mock stained or stained with human Fcγ fragment-FITC (Rockland Immunochemicals, USA). 1×10^4^ living cells were obtained in FACSCanto II using the FACS Diva software and analyzed with FLowJo (Tree Star Inc, USA).

### FACS analysis for HCMV and HSV surface antigen expression on infected cells

MRC-5 cells were infected with 1 PFU/cell of wt HCMV and HCMV ΔvFcγR mutants for 72 h and with 10 PFU/cell of HSV-1 wt and ΔgE for 24 h. Cells were resuspended in PBS containing 2 mM EDTA, washed twice in PBS supplemented with 3% (vol/vol) FCS. HCMV infected cells were stained with the F(ab)_2_ preparation of Cytotect, goat anti-human-F(ab)_2_-Biotin, and Streptavidin-PE (AdB Serotec, UK) or Fcγ fragment-FITC (Rockland Immunochemicals, USA). The comparability of infection of the different HCMV ΔvFcγR mutants was controlled by intracellular staining of CMV nuclear antigens with CCH2 and DDG9 antibodies (Dako, Denmark) and goat anti-mouse-APC (BD Pharmingen, USA) after fixation with 1,5% PFA and permeabilization with PBS supplemented with 3% (vol/vol) FCS and 0,05% (vol/vol) Saponin. HSV infected cells were stained with the F(ab)_2_ preparation of Cytotect, goat anti-human-F(ab)_2_-Biotin (AdB Serotec, UK) or Fcγ fragment-Biotin (Rockland Immunochemicals, USA) and Streptavidin-APC (Jackson Immunoresearch, USA). After DAPI staining, 1–2×10^4^ living cells were obtained in a FACSCanto II using the FACS Diva software and analyzed with FlowJo (Tree Star Inc, USA).

### CD107a NK cell degranulation assay

PBMCs were prepared from EDTA-blood of healthy donors using Lymphoprep (Axis-Shield, Norway) differential centrifugation. PBMCs were incubated during 3 h at 37°C to allow adherence of unwanted cells. Suspension cells were collected and resuspended in media containing 100 IU/ml of human rIL-2 (PromoKine, Germany) and incubated overnight at 37°C. Cells were resuspended and further processed to obtained polyclonal NK cells using a MACS negative selection NK cell isolation kit (Miltenyi Biotec, Germany). NK cell purity was tested in FACS and was usually above 96% (data not shown). For measuring degranulation by co-cultivation of immune IgG and a viral target, HCMV or HSV infected fibroblasts were opsonized with a serum of a healthy donor positive for HCMV and HSV or with IVIG. As a control, a healthy seronegative donor was also analyzed. Opsonization was done at 37°C for 30 min at 5% CO_2_. Two steps of washing with D-MEM 10% (vol/vol) FCS followed to remove unbound IgG. 1×10^5^ polyclonal NK cells (E∶T ratio of 10∶1) were added in each well and the CD107a assay was performed as elsewhere described [43]. Briefly, polyclonal human NK cells were incubated 4 h at 37°C in the presence of 6 µg/ml Golgi Stop (Monensin, BD Pharmingen, Belgium), 10 µg/ml Golgi Plug (BrefeldinA, BD Pharmingen, Belgium), and CD107-PeCy5 mAb (BD Pharmigen, Belgium). NK cells were collected, washed twice in ice cold PBS containing 2 mM EDTA and stained for extracellular markers (CD56, CD3). 1×10^4^ cells were counted and analyzed.

### Immunoprecipitation and detection of proteins by immunoblot

SKOV-3 cells were infected with 2 PFU/cell VACV for 14 h. Infected cells were incubated with 1 µg/ml trastuzumab or palivizumab for 30 min at 4°C, and non-bound antibody was removed by washing cells with PBS. Lysis buffer (200 mM NaCl, 10 mM MgCl_2_, 10 mM KCl, 20 mM HEPES, 0.5% (vol/vol) NP-40, 0.1M EDTA, 10% (vol/vol) glycerol, 0.1 mM sodium orthovanadate, 0.1 mM PMSF, 0.5 µM Pepstatin, 1 mM dithiothreitol, pH 7.4) was given to cells and, after removal of cell nuclei by centrifugation, lysates were incubated with agarose immobilized anti-FLAG antibody (Bethyl Laboratories, Inc. USA) during 2 h at 4°C. Previously, a sample of each lysate was taken for subsequent western blot expression analysis. Lysis buffer was used to wash the agarose pellet and proteins were eluted with Laemmli sample buffer. Proteins were separated by sodium-dodecyl-sulfate (SDS)-8% polyacrylamide gel electrophoresis (PAGE) and transferred to nitrocellulose filters. Western Blot was performed with anti-ErbB2-specific rabbit mAb V2W (Abcam Inc, USA), anti-Flag-specific mouse mAb M2 (Sigma-Aldrich, USA), anti-human-peroxidase (Sigma-Aldrich, USA), anti-rabbit-peroxidase (Sigma-Aldrich, USA) and anti-mouse-peroxidase (Dianova, Germany). Proteins were visualized using ECL chemiluminescence system (GE-Healthcare, Germany).

### Uniprot list of genes and proteins used

HCMV UL119-118: **P16739**

HCMV RL11: **Q6SX56**

HSV gE: **Q703F0**

Human FcγRIIIA/CD16: **P08637**

Human FcγRIIA/CD32A: **P12318**

Human FcγRI/CD64: **P12314**

Mouse TCR zeta chain: **P24161**

Human ICOSL: **O75144**

### Taxonomy ID for viruses

Human cytomegalovirus (strain AD169): **10360**

Herpes simplex virus (type 1/strain F): **10304**

Vaccinia virus Western Reserve: **696871**

## Supporting Information

Figure S1 **BW:FcγR-ζ responses are virus-specific and triggered only in the presence of virus-immune IgG.** MRC-5 cells were infected with 2 PFU/cell HSV-1 wt, HCMV HB5 wt or left uninfected (mock) for 24 or 72 h. Afterwards, cells were opsonized with IgG of pooled human sera. ELISA-reactive (immune) HSV IgG and HCMV IgG sera were compared with ELISA non-reactive (non-immune) sera at 2 mg/ml of IgG concentration. After washing, BW:FcγR-ζ effector cells were added and cultures were incubated for 16 h. mIL-2 was determined by ELISA. Triplicates were measured; means with standard deviations (error bars) are shown for 2 independent experiments. n.d. indicates OD<0.05.
(TIF)

Figure S2 **Detection of HCMV and HSV surface antigen expression on infected cells.** (**A**) MRC-5 cells were infected with HSV-1 wt, ΔgE and ΔgE-revertant with 2 PFU/cell for 24 h. After harvesting and washing in PBS with 3% (vol/vol) FCS cells were mock stained, stained with human Fcγ-FITC or stained with a purified F(ab)_2_ preparation of Cytotect, followed by goat anti-human-F(ab)_2_-Biotin and Streptavidin-PE. 1×10^4^ living cells were analyzed with a FACSCanto II using the FACS Diva software and analyzed with FLowJo (Tree Star Inc, USA). (**B**) As in (A), but MRC- 5 fibroblasts were infected with HCMV HB5 wt or HB5Δgp68 with 2 PFU/cell for 72 h. (**C**) as in (B), but MRC-5 cells were infected with HB5ΔIRL, HB5ΔIRLΔgp34 or HB5ΔIRLΔgp34/Δgp68. (**D**) As in (B), but MRC-5 cells were infected with AD169varL wt, AD169varLΔgp68, AD169varLΔgp34 or AD169varLΔgp34/Δgp68. One of three (A, B, C) or two (D) representative experiments is shown.
(TIF)

Figure S3 **HCMV TB40/E BACmid derived vFcγR revertants restore FcγRIIIA inhibition.** MRC-5 cells were infected with HCMV wt virus, vFcγR mutants or vFcγR revertants (2 PFU/cell) for 96 h. (**A**) Infected MRC-5 fibroblasts were stained with purified F(ab)_2_ fragments prepared from IVIG Cytotect, Fcγ-FITC or 2^nd^ step antibody as a control and analysed by FACS. (**B**) MRC-5 fibroblasts were opsonized with IVIG Cytotect at different concentrations for 30 min. After removing of unbound antibodies by washing, 1×10^5^ BW:FcγR-ζ transfectants were added. Measurement of mIL-2 in supernatants after 16 h of co-cultivation of reporter cells with targets was performed by ELISA. Values are presented in the graphic as OD 450 nm. n = 3; means with standard deviations (error bars) are shown for two independent experiments.
(TIF)

Figure S4 **Ectopic expression of HSV-1 gE, HCMV gp68 and HCMV gp34 inhibit IgG1 mediated activation of FcγRIIA.** CD20 transfected 293T cells were infected for 16 hours with 2 PFU/cell of VACV wt or rVACV expressing gE (**A**) or gp68 and gp34 (**B**). After opsonization with 4 µg of rituximab (anti-hCD20 IgG1) and washing for removing unbound antibody, cells were co-cultivated with 1×10^5^ BW:FcγRIIA-ζ reporter cells per well for 16 h before supernatants were collected and mIL-2 was determined by ELISA. Each value represents three replicates; means with standard deviations (error bars) are shown for two independent experiments. Significance of results (Student's t-test) are presented in Table S1 as *: p<0.05 **: p<0.01 ***: p<0.001.
(TIF)

Figure S5 **Detection of soluble vFcγRs ectodomains.** To compare amounts of soluble proteins used in the BW:FcγR-ζ assay, recombinant proteins were loaded in different dilution steps on an SDS-PAGE and detected using an anti-V5 antibody by western blot. Due to the strong inhibition capacity of sgp34 protein at very low concentrations, its amounts are hardly detectable in the blot. Therefore higher concentrations (200×, 100×) and a longer exposure are shown.
(TIF)

Table S1 Significance of results (Student's t-test) is presented in Table S1 as *: p<0.05 **: p<0.01 ***: p<0.001 for all figures in need of it.
(DOCX)

Table S2 Synopsis of HCMV mutants used in the study.
(DOCX)

## References

[1] Mocarski,E.S., Shenk,T., and Pass RF (2007) Cytomegaloviruses. In: Knipe, D.K and Howley PM, editor. Field's Virology. Philadephia: Lippincott, Williams & Wilkins. pp. 2701–2772.

[2] Stern-Ginossar N, Weisburd B, Michalski A, Le VTK, Hein MY, et al (2012) Decoding human cytomegalovirus. Science 338: 1088–1093. 23180859 10.1126/science.1227919 PMC3817102

[3] Quinnan G V, Masur H, Rook AH, Armstrong G, Frederick WR, et al (1984) Herpesvirus infections in the acquired immune deficiency syndrome. JAMA 252: 72–77. 6328055

[4] Polić B, Hengel H, Krmpotić A, Trgovcich J, Pavić I, et al (1998) Hierarchical and redundant lymphocyte subset control precludes cytomegalovirus replication during latent infection. J Exp Med 188: 1047–1054. 9743523 10.1084/jem.188.6.1047 PMC2212537

[5] Miller-Kittrell M, Sparer TE (2009) Feeling manipulated: cytomegalovirus immune manipulation. Virol J 6: 4. 19134204 10.1186/1743-422X-6-4 PMC2636769

[6] Mocarski ES (2002) Immunomodulation by cytomegaloviruses: manipulative strategies beyond evasion. Trends Microbiol 10: 332–339. 12110212 10.1016/s0966-842x(02)02393-4

[7] Mocarski ES (2004) Immune escape and exploitation strategies of cytomegaloviruses: impact on and imitation of the major histocompatibility system. Cell Microbiol 6: 707–717. 15236638 10.1111/j.1462-5822.2004.00425.x

[8] Jonjić S, Pavić I, Polić B, Crnković I, Lucin P, et al (1994) Antibodies are not essential for the resolution of primary cytomegalovirus infection but limit dissemination of recurrent virus. J Exp Med 179: 1713–1717. 8163949 10.1084/jem.179.5.1713 PMC2191473

[9] Klenovsek K, Weisel F, Schneider A, Appelt U, Jonjic S, et al (2007) Protection from CMV infection in immunodeficient hosts by adoptive transfer of memory B cells. Blood 110: 3472–3479. 17656648 10.1182/blood-2007-06-095414

[10] Cekinović D, Golemac M, Pugel EP, Tomac J, Cicin-Sain L, et al (2008) Passive immunization reduces murine cytomegalovirus-induced brain pathology in newborn mice. J Virol 82: 12172–12180. 18842707 10.1128/JVI.01214-08 PMC2593357

[11] Nigro G, Adler SP, La Torre R, Best AM (2005) Passive immunization during pregnancy for congenital cytomegalovirus infection. N Engl J Med 353: 1350–1362. 16192480 10.1056/NEJMoa043337

[12] Raanani P, Gafter-Gvili A, Paul M, Ben-Bassat I, Leibovici L, et al (2009) Immunoglobulin prophylaxis in hematopoietic stem cell transplantation: systematic review and meta-analysis. J Clin Oncol 27: 770–781. 19114702 10.1200/JCO.2008.16.8450

[13] Hodson EM, Jones CA, Strippoli GFM, Webster AC, Craig JC (2007) Immunoglobulins, vaccines or interferon for preventing cytomegalovirus disease in solid organ transplant recipients. Cochrane database Syst Rev CD005129. 17443573 10.1002/14651858.CD005129.pub2 PMC13199907

[14] Boeckh M, Bowden RA, Storer B, Chao NJ, Spielberger R, et al (2001) Randomized, placebo-controlled, double-blind study of a cytomegalovirus-specific monoclonal antibody (MSL-109) for prevention of cytomegalovirus infection after allogeneic hematopoietic stem cell transplantation. Biol Blood Marrow Transplant 7: 343–351. 11464977 10.1016/s1083-8791(01)80005-7

[15] Bruhns P, Iannascoli B, England P, Mancardi DA, Fernandez N, et al (2009) Specificity and affinity of human Fcgamma receptors and their polymorphic variants for human IgG subclasses. Blood 113: 3716–3725. 19018092 10.1182/blood-2008-09-179754

[16] Nimmerjahn F, Ravetch JV (2007) Fc-receptors as regulators of immunity. Adv Immunol 96: 179–204. 17981207 10.1016/S0065-2776(07)96005-8

[17] Bruhns P (2012) Properties of mouse and human IgG receptors and their contribution to disease models. Blood 119: 5640–5649. 22535666 10.1182/blood-2012-01-380121

[18] Ernst LK, Metes D, Herberman RB, Morel PA (2002) Allelic polymorphisms in the FcgammaRIIC gene can influence its function on normal human natural killer cells. J Mol Med (Berl) 80: 248–257. 11976734 10.1007/s00109-001-0294-2

[19] Metes D, Manciulea M, Pretrusca D, Rabinowich H, Ernst LK, et al (1999) Ligand binding specificities and signal transduction pathways of Fc gamma receptor IIc isoforms: the CD32 isoforms expressed by human NK cells. Eur J Immunol 29: 2842–2852. 10508259 10.1002/(SICI)1521-4141(199909)29:09<2842::AID-IMMU2842>3.0.CO;2-5

[20] Furukawa T, Hornberger E, Sakuma S, Plotkin SA (1975) Demonstration of immunoglobulin G receptors induced by human cytomegalovirus. J Clin Microbiol 2: 332–336. 171279 10.1128/jcm.2.4.332-336.1975 PMC362805

[21] Budt M, Reinhard H, Bigl A, Hengel H (2004) Herpesviral Fcgamma receptors: culprits attenuating antiviral IgG? Int Immunopharmacol 4: 1135–1148. 15251110 10.1016/j.intimp.2004.05.020 PMC7173100

[22] Atalay R, Zimmermann A, Wagner M, Borst E, Benz C, et al (2002) Identification and expression of human cytomegalovirus transcription units coding for two distinct Fcgamma receptor homologs. J Virol 76: 8596–8608. 12163579 10.1128/JVI.76.17.8596-8608.2002 PMC136976

[23] Lilley BN, Ploegh HL, Tirabassi RS (2001) Human cytomegalovirus open reading frame TRL11/IRL11 encodes an immunoglobulin G Fc-binding protein. J Virol 75: 11218–11221. 11602761 10.1128/JVI.75.22.11218-11221.2001 PMC114701

[24] Sprague ER, Reinhard H, Cheung EJ, Farley AH, Trujillo RD, et al (2008) The human cytomegalovirus Fc receptor gp68 binds the Fc CH2-CH3 interface of immunoglobulin G. J Virol 82: 3490–3499. 18216124 10.1128/JVI.01476-07 PMC2268481

[25] Kutza AS, Muhl E, Hackstein H, Kirchner H, Bein G (1998) High incidence of active cytomegalovirus infection among septic patients. Clin Infect Dis 26: 1076–1082. 9597229 10.1086/520307

[26] Ross SA, Arora N, Novak Z, Fowler KB, Britt WJ, et al (2010) Cytomegalovirus reinfections in healthy seroimmune women. J Infect Dis 201: 386–389. 20039807 10.1086/649903 PMC2804773

[27] Hansen SG, Powers CJ, Richards R, Ventura AB, Ford JC, et al (2010) Evasion of CD8+ T cells is critical for superinfection by cytomegalovirus. Science 328: 102–106. 20360110 10.1126/science.1185350 PMC2883175

[28] Corrales-Aguilar E, Trilling M, Reinhard H, Mercé-Maldonado E, Widera M, et al (2013) A novel assay for detecting virus-specific antibodies triggering activation of Fcγ receptors. J Immunol Methods 387: 21–35. 23023090 10.1016/j.jim.2012.09.006

[29] Johnson DC, Frame MC, Ligas MW, Cross AM, Stow ND (1988) Herpes simplex virus immunoglobulin G Fc receptor activity depends on a complex of two viral glycoproteins, gE and gI. J Virol 62: 1347–1354. 2831396 10.1128/jvi.62.4.1347-1354.1988 PMC253147

[30] Dubin G, Socolof E, Frank I, Friedman HM (1991) Herpes simplex virus type 1 Fc receptor protects infected cells from antibody-dependent cellular cytotoxicity. J Virol 65: 7046–7050. 1658396 10.1128/jvi.65.12.7046-7050.1991 PMC250825

[31] Halenius A, Hauka S, Dölken L, Stindt J, Reinhard H, et al (2011) Human cytomegalovirus disrupts the major histocompatibility complex class I peptide-loading complex and inhibits tapasin gene transcription. J Virol 85: 3473–3485. 21248040 10.1128/JVI.01923-10 PMC3067861

[32] Hoetzenecker K, Hacker S, Hoetzenecker W, Sadeghi K, Sachet M, et al (2007) Cytomegalovirus hyperimmunoglobulin: mechanisms in allo-immune response in vitro. Eur J Clin Invest 37: 978–986. 18036032 10.1111/j.1365-2362.2007.01881.x

[33] Le VTK, Trilling M, Hengel H (2011) The cytomegaloviral protein pUL138 acts as potentiator of tumor necrosis factor (TNF) receptor 1 surface density to enhance ULb'-encoded modulation of TNF-α signaling. J Virol 85: 13260–13270. 21976655 10.1128/JVI.06005-11 PMC3233134

[34] Sinzger C, Hahn G, Digel M, Katona R, Sampaio KL, et al (2008) Cloning and sequencing of a highly productive, endotheliotropic virus strain derived from human cytomegalovirus TB40/E. J Gen Virol 89: 359–368. 18198366 10.1099/vir.0.83286-0

[35] Morizono K, Ku A, Xie Y, Harui A, Kung SKP, et al (2010) Redirecting lentiviral vectors pseudotyped with Sindbis virus-derived envelope proteins to DC-SIGN by modification of N-linked glycans of envelope proteins. J Virol 84: 6923–6934. 20484510 10.1128/JVI.00435-10 PMC2898243

[36] Linde GA, Hammarström L, Persson MA, Smith CI, Sundqvist VA, et al (1983) Virus-specific antibody activity of different subclasses of immunoglobulins G and A in cytomegalovirus infections. Infect Immun 42: 237–244. 6311746 10.1128/iai.42.1.237-244.1983 PMC264549

[37] Gupta CK, Leszczynski J, Gupta RK, Siber GR (1996) IgG subclass antibodies to human cytomegalovirus (CMV) in normal human plasma samples and immune globulins and their neutralizing activities. Biologicals 24: 117–124. 8889058 10.1006/biol.1996.0015

[38] Wiger D, Michaelsen TE (1985) Binding site and subclass specificity of the herpes simplex virus type 1-induced Fc receptor. Immunology 54: 565–572. 2982735 PMC1453521

[39] Johansson PJ, Hallberg T, Oxelius VA, Grubb A, Blomberg J (1984) Human immunoglobulin class and subclass specificity of Fc receptors induced by herpes simplex virus type 1. J Virol 50: 796–804. 6328009 10.1128/jvi.50.3.796-804.1984 PMC255739

[40] Mandelboim O, Malik P, Davis DM, Jo CH, Boyson JE, et al (1999) Human CD16 as a lysis receptor mediating direct natural killer cell cytotoxicity. Proc Natl Acad Sci U S A 96: 5640–5644. 10318937 10.1073/pnas.96.10.5640 PMC21913

[41] Cooper MA, Fehniger TA, Caligiuri MA (2001) The biology of human natural killer-cell subsets. Trends Immunol 22: 633–640. 11698225 10.1016/s1471-4906(01)02060-9

[42] Couzi L, Pitard V, Sicard X, Garrigue I, Hawchar O, et al (2012) Antibody-dependent anti-cytomegalovirus activity of human γδ T cells expressing CD16 (FcγRIIIa). Blood 119: 1418–1427. 22180442 10.1182/blood-2011-06-363655

[43] Alter G, Malenfant JM, Altfeld M (2004) CD107a as a functional marker for the identification of natural killer cell activity. J Immunol Methods 294: 15–22. 15604012 10.1016/j.jim.2004.08.008

[44] Gumá M, Angulo A, López-Botet M (2006) NK cell receptors involved in the response to human cytomegalovirus infection. Curr Top Microbiol Immunol 298: 207–223. 16323417 10.1007/3-540-27743-9_11

[45] Wilkinson GWG, Tomasec P, Stanton RJ, Armstrong M, Prod'homme V, et al (2008) Modulation of natural killer cells by human cytomegalovirus. J Clin Virol 41: 206–212. 18069056 10.1016/j.jcv.2007.10.027 PMC2843162

[46] Lenac T, Budt M, Arapovic J, Hasan M, Zimmermann A, et al (2006) The herpesviral Fc receptor fcr-1 down-regulates the NKG2D ligands MULT-1 and H60. J Exp Med 203: 1843–1850. 16831899 10.1084/jem.20060514 PMC2118374

[47] Frank I, Friedman HM (1989) A novel function of the herpes simplex virus type 1 Fc receptor: participation in bipolar bridging of antiviral immunoglobulin G. J Virol 63: 4479–4488. 2552134 10.1128/jvi.63.11.4479-4488.1989 PMC251078

[48] Van Vliet KE, De Graaf-Miltenburg LA, Verhoef J, Van Strijp JA (1992) Direct evidence for antibody bipolar bridging on herpes simplex virus-infected cells. Immunology 77: 109–115. 1328043 PMC1421581

[49] Sprague ER, Wang C, Baker D, Bjorkman PJ (2006) Crystal structure of the HSV-1 Fc receptor bound to Fc reveals a mechanism for antibody bipolar bridging. PLoS Biol 4: e148. 16646632 10.1371/journal.pbio.0040148 PMC1450327

[50] Sprague ER, Martin WL, Bjorkman PJ (2004) pH dependence and stoichiometry of binding to the Fc region of IgG by the herpes simplex virus Fc receptor gE-gI. J Biol Chem 279: 14184–14193. 14734541 10.1074/jbc.M313281200

[51] Schoppel K, Kropff B, Schmidt C, Vornhagen R, Mach M (1997) The humoral immune response against human cytomegalovirus is characterized by a delayed synthesis of glycoprotein-specific antibodies. J Infect Dis 175: 533–544. 9041323 10.1093/infdis/175.3.533

[52] Lubinski J, Nagashunmugam T, Friedman HM (1998) Viral interference with antibody and complement. Semin Cell Dev Biol 9: 329–337. 9665870 10.1006/scdb.1998.0242 PMC7172161

[53] Nagashunmugam T, Lubinski J, Wang L, Goldstein LT, Weeks BS, et al (1998) In vivo immune evasion mediated by the herpes simplex virus type 1 immunoglobulin G Fc receptor. J Virol 72: 5351–5359. 9620988 10.1128/jvi.72.7.5351-5359.1998 PMC110157

[54] Lubinski JM, Lazear HM, Awasthi S, Wang F, Friedman HM (2011) The herpes simplex virus 1 IgG fc receptor blocks antibody-mediated complement activation and antibody-dependent cellular cytotoxicity in vivo. J Virol 85: 3239–3249. 21228231 10.1128/JVI.02509-10 PMC3067879

[55] Sondermann P, Huber R, Oosthuizen V, Jacob U (2000) The 3.2-A crystal structure of the human IgG1 Fc fragment-Fc gammaRIII complex. Nature 406: 267–273. 10917521 10.1038/35018508

[56] Radaev S, Motyka S, Fridman WH, Sautes-Fridman C, Sun PD (2001) The structure of a human type III Fcgamma receptor in complex with Fc. J Biol Chem 276: 16469–16477. 11297532 10.1074/jbc.M100350200

[57] Sondermann P, Oosthuizen V (2002) X-ray crystallographic studies of IgG-Fc gamma receptor interactions. Biochem Soc Trans 30: 481–486. 12196119 10.1042/bst0300481

[58] Roux KH, Strelets L, Michaelsen TE (1997) Flexibility of human IgG subclasses. J Immunol 159: 3372–3382. 9317136

[59] Hengel H, Brune W, Koszinowski UH (1998) Immune evasion by cytomegalovirus–survival strategies of a highly adapted opportunist. Trends Microbiol 6: 190–197. 9614343 10.1016/s0966-842x(98)01255-4

[60] Powers C, Früh K (2008) Rhesus CMV: an emerging animal model for human CMV. Med Microbiol Immunol 197: 109–115. 18193454 10.1007/s00430-007-0073-y PMC2746484

[61] Jonjić S, Babić M, Polić B, Krmpotić A (2008) Immune evasion of natural killer cells by viruses. Curr Opin Immunol 20: 30–38. 18206359 10.1016/j.coi.2007.11.002 PMC2700287

[62] Hengel H, Koszinowski UH (1997) Interference with antigen processing by viruses. Curr Opin Immunol 9: 470–476. 9287180 10.1016/s0952-7915(97)80097-0

[63] Arapovic J, Lenac T, Antulov R, Polic B, Ruzsics Z, et al (2009) Differential susceptibility of RAE-1 isoforms to mouse cytomegalovirus. J Virol 83: 8198–8207. 19494006 10.1128/JVI.02549-08 PMC2715744

[64] Mintern JD, Klemm EJ, Wagner M, Paquet ME, Napier MD, et al (2006) Viral interference with B7-1 costimulation: a new role for murine cytomegalovirus fc receptor-1. J Immunol 177: 8422–8431. 17142739 10.4049/jimmunol.177.12.8422

[65] Cortese M, Calò S, D'Aurizio R, Lilja A, Pacchiani N, et al (2012) Recombinant human cytomegalovirus (HCMV) RL13 binds human immunoglobulin G Fc. PLoS One 7: e50166. 23226246 10.1371/journal.pone.0050166 PMC3511460

[66] Khan N (n.d.) The immunological burden of human cytomegalovirus infection. Arch Immunol Ther Exp (Warsz) 55: 299–308. 18219760 10.1007/s00005-007-0037-3

[67] Radsak K, Eickmann M, Mockenhaupt T, Bogner E, Kern H, et al (1996) Retrieval of human cytomegalovirus glycoprotein B from the infected cell surface for virus envelopment. Arch Virol 141: 557–572. 8645095 10.1007/BF01718317

[68] Manley K, Anderson J, Yang F, Szustakowski J, Oakeley EJ, et al (2011) Human cytomegalovirus escapes a naturally occurring neutralizing antibody by incorporating it into assembling virions. Cell Host Microbe 10: 197–209. 21925108 10.1016/j.chom.2011.07.010

[69] Macagno A, Bernasconi NL, Vanzetta F, Dander E, Sarasini A, et al (2010) Isolation of human monoclonal antibodies that potently neutralize human cytomegalovirus infection by targeting different epitopes on the gH/gL/UL128-131A complex. J Virol 84: 1005–1013. 19889756 10.1128/JVI.01809-09 PMC2798344

[70] Plachter B, Sinzger C, Jahn G (1996) Cell types involved in replication and distribution of human cytomegalovirus. Adv Virus Res 46: 195–261. 8824701 10.1016/s0065-3527(08)60073-1

[71] Borst EM, Hahn G, Koszinowski UH, Messerle M (1999) Cloning of the human cytomegalovirus (HCMV) genome as an infectious bacterial artificial chromosome in Escherichia coli: a new approach for construction of HCMV mutants. J Virol 73: 8320–8329. 10482582 10.1128/jvi.73.10.8320-8329.1999 PMC112849

[72] Dingwell KS, Brunetti CR, Hendricks RL, Tang Q, Tang M, et al (1994) Herpes simplex virus glycoproteins E and I facilitate cell-to-cell spread in vivo and across junctions of cultured cells. J Virol 68: 834–845. 8289387 10.1128/jvi.68.2.834-845.1994 PMC236520

[73] Bell S, Cranage M, Borysiewicz L, Minson T (1990) Induction of immunoglobulin G Fc receptors by recombinant vaccinia viruses expressing glycoproteins E and I of herpes simplex virus type 1. J Virol 64: 2181–2186. 2157879 10.1128/jvi.64.5.2181-2186.1990 PMC249377

[74] Krmpotic A, Hasan M, Loewendorf A, Saulig T, Halenius A, et al (2005) NK cell activation through the NKG2D ligand MULT-1 is selectively prevented by the glycoprotein encoded by mouse cytomegalovirus gene m145. J Exp Med 201: 211–220. 15642742 10.1084/jem.20041617 PMC2212792

[75] Wagner M, Gutermann A, Podlech J, Reddehase MJ, Koszinowski UH (2002) Major histocompatibility complex class I allele-specific cooperative and competitive interactions between immune evasion proteins of cytomegalovirus. J Exp Med 196: 805–816. 12235213 10.1084/jem.20020811 PMC2194048

[76] Messerle M, Crnkovic I, Hammerschmidt W, Ziegler H, Koszinowski UH (1997) Cloning and mutagenesis of a herpesvirus genome as an infectious bacterial artificial chromosome. Proc Natl Acad Sci U S A 94: 14759–14763. 9405686 10.1073/pnas.94.26.14759 PMC25110

[77] Snydman DR, Werner BG, Heinze-Lacey B, Berardi VP, Tilney NL, et al (1987) Use of cytomegalovirus immune globulin to prevent cytomegalovirus disease in renal-transplant recipients. N Engl J Med 317: 1049–1054. 2821397 10.1056/NEJM198710223171703

