# Supplementary figures and images for: Human Cytomegalovirus Fcγ Binding Proteins gp34 and gp68 Antagonize Fcγ Receptors I, II and III
Source: PLoS Pathog. 2014 May 15;10(5):e1004131. doi: 10.1371/journal.ppat.1004131 (PMC4022731; doi:10.1371/journal.ppat.1004131)

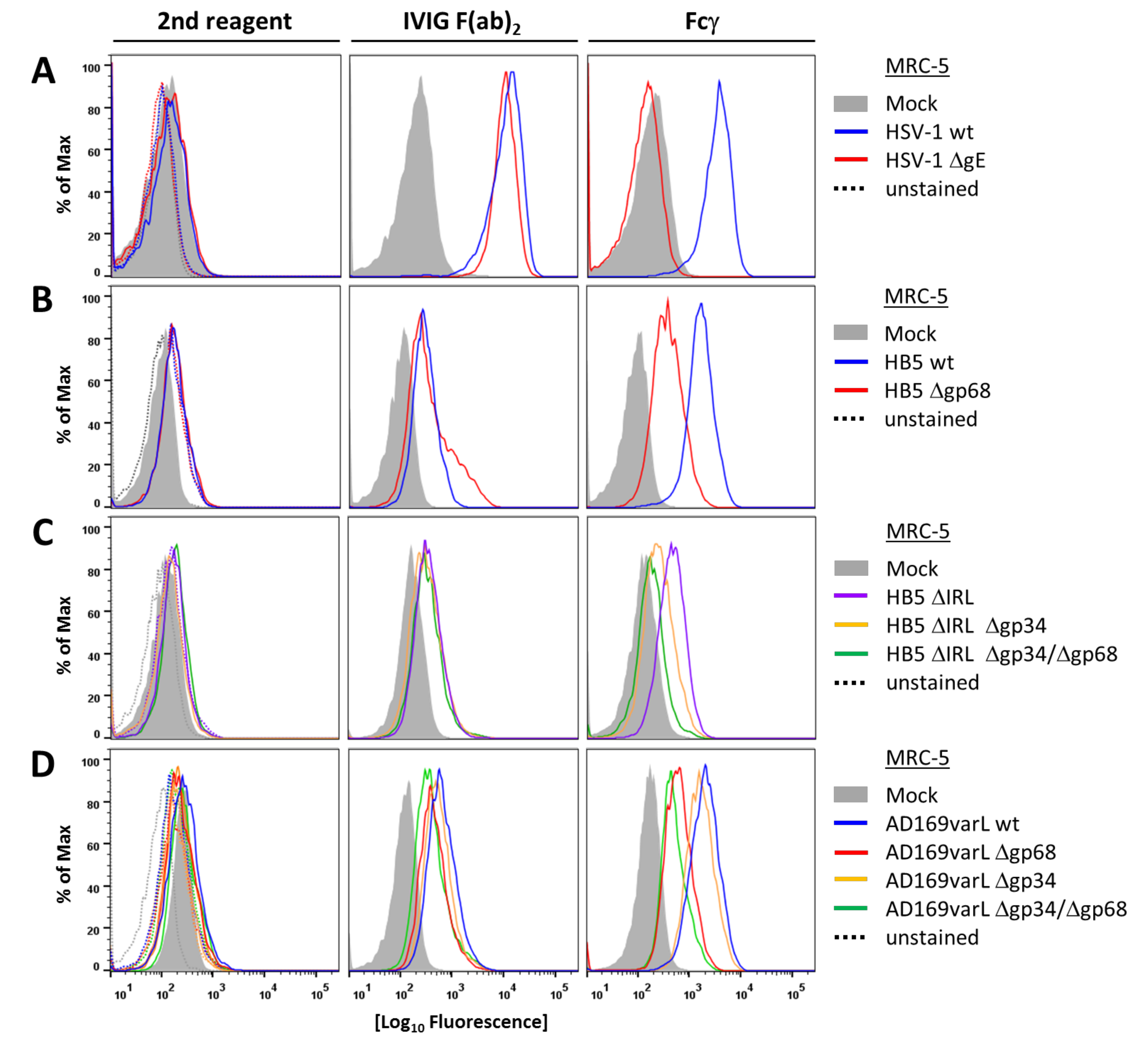

Supplement: Figure S2 — Detection of HCMV and HSV surface antigen expression on infected cells. (A) MRC-5 cells were infected with HSV-1 wt, ΔgE and ΔgE-revertant with 2 PFU/cell for 24 h. After harvesting and washing in PBS with 3% (vol/vol) FCS cells were mock stained, stained with human Fcγ-FITC or stained with a purified F(ab)2 preparation of Cytotect, followed by goat anti-human-F(ab)2-Biotin and Streptavidin-PE. 1×10^4^ living cells were analyzed with a FACSCanto II using the FACS Diva software and analyzed with FLowJo (Tree Star Inc, USA). (B) As in (A), but MRC- 5 fibroblasts were infected with HCMV HB5 wt or HB5Δgp68 with 2 PFU/cell for 72 h. (C) as in (B), but MRC-5 cells were infected with HB5ΔIRL, HB5ΔIRLΔgp34 or HB5ΔIRLΔgp34/Δgp68. (D) As in (B), but MRC-5 cells were infected with AD169varL wt, AD169varLΔgp68, AD169varLΔgp34 or AD169varLΔgp34/Δgp68. One of three (A, B, C) or two (D) representative experiments is shown. (TIF) [file ppat.1004131.s002.tif]

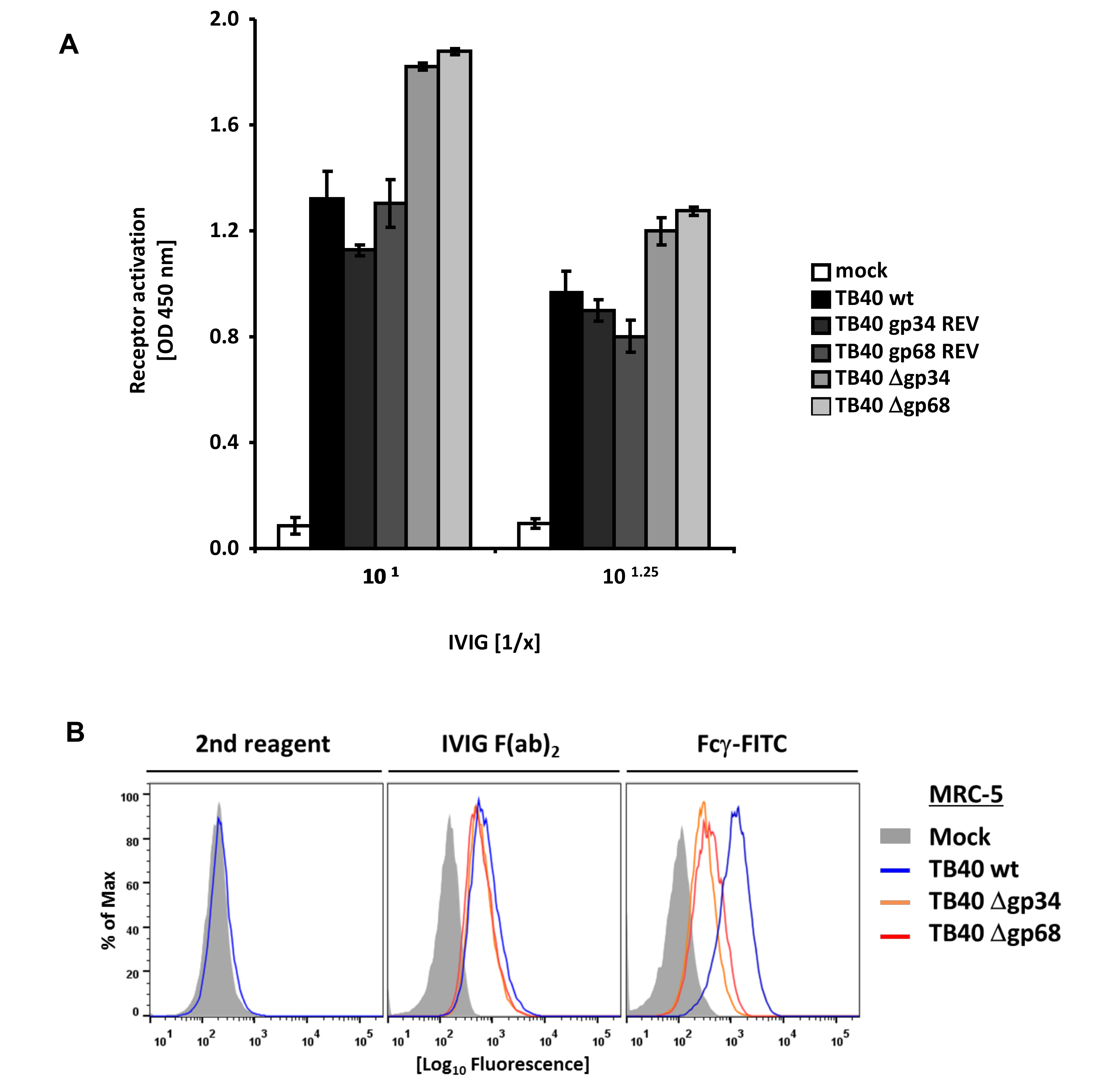

Supplement: Figure S3 — HCMV TB40/E BACmid derived vFcγR revertants restore FcγRIIIA inhibition. MRC-5 cells were infected with HCMV wt virus, vFcγR mutants or vFcγR revertants (2 PFU/cell) for 96 h. (A) Infected MRC-5 fibroblasts were stained with purified F(ab)2 fragments prepared from IVIG Cytotect, Fcγ-FITC or 2nd step antibody as a control and analysed by FACS. (B) MRC-5 fibroblasts were opsonized with IVIG Cytotect at different concentrations for 30 min. After removing of unbound antibodies by washing, 1×10^5^ BW:FcγR-ζ transfectants were added. Measurement of mIL-2 in supernatants after 16 h of co-cultivation of reporter cells with targets was performed by ELISA. Values are presented in the graphic as OD 450 nm. n = 3; means with standard deviations (error bars) are shown for two independent experiments. (TIF) [file ppat.1004131.s003.tif]
