# Supplementary material for: Human Cytomegalovirus Fcγ Binding Proteins gp34 and gp68 Antagonize Fcγ Receptors I, II and III
Source: PLoS Pathog. 2014 May 15;10(5):e1004131. doi: 10.1371/journal.ppat.1004131 (PMC4022731; doi:10.1371/journal.ppat.1004131)
Supplement: Table S1 — Significance of results (Student's t-test) is presented in Table S1 as *: p<0.05 **: p<0.01 ***: p<0.001 for all figures in need of it. (DOCX) [file ppat.1004131.s006.docx]

| Table S1 |  |  |  |  |  |  |  |  |
| --- | --- | --- | --- | --- | --- | --- | --- | --- |
| **p-Values** | **> 0.05** | **< 0.05** | **< 0.01** | **< 0.001** |  |  |  |  |
| **Significance** | **n.s.** | ***** | ****** | ******* |  |  |  |  |
| **Against wt or sICOSL** | **n.s.= not significant** | |  |  |  |  |  |  |
|  | **n.a.=not activated** | |  |  |  |  |  |  |
|  |  |  |  |  |  |  |  |  |
| **Figure 1B** | IVIG Dilution (1/X) | |  |  |  |  |  |  |
|  | 10 | 20 | 40 | 80 |  |  |  |  |
| with PAA | *** | *** | n.s. | n.s. |  |  |  |  |
|  |  |  |  |  |  |  |  |  |
| **Figure 1C** | IVIG Dilution (1/X) | |  |  |  |  |  |  |
|  | 32 | 64 | 128 | 256 | 512 | 1024 |  |  |
| with PAA | *** | ** | * | n.s. | n.s. | n.s. |  |  |
|  |  |  |  |  |  |  |  |  |
| **Figure 2A** | IVIG Dilution (1/X) | |  |  |  |  |  |  |
| **FcγRIIIA-ζ** | 128 | 256 | 512 | 1024 | 2048 | 4096 | 8192 |  |
| ΔgE | *** | ** | * | ** | n.s. | n.s. | n.s. |  |
|  |  |  |  |  |  |  |  |  |
|  | IVIG Dilution (1/X) | |  |  |  |  |  |  |
| **FcγRIIA-ζ** | 128 | 256 | 512 | 1024 | 2048 | 4096 | 8192 |  |
| ΔgE | * | *** | *** | *** | * | n.s. | n.s. |  |
|  |  |  |  |  |  |  |  |  |
|  | IVIG Dilution (1/X) | |  |  |  |  |  |  |
| **FcγRI-ζ** | 128 | 256 | 512 | 1024 | 2048 | 4096 | 8192 |  |
| ΔgE | n.s. | n.s. | *** | ** | ** | n.s. | n.s. |  |
|  |  |  |  |  |  |  |  |  |
| **Figure 2B** | IVIG Dilution (1/X) | |  |  |  |  |  |  |
| **FcγRIIIA-ζ** | 8 | 16 | 32 | 64 | 128 | 256 | 512 | 1024 |
| HB5 Δgp68 | ** | * | * | * | * | *** | *** | n.s. |
|  |  |  |  |  |  |  |  |  |
|  | IVIG Dilution (1/X) | |  |  |  |  |  |  |
| **FcγRIIA-ζ** | 8 | 16 | 32 | 64 | 128 | 256 | 512 | 1024 |
| HB5 Δgp68 | *** | *** | ** | *** | ** | *** | * | * |
|  |  |  |  |  |  |  |  |  |
|  | IVIG Dilution (1/X) | |  |  |  |  |  |  |
| **FcγRI-ζ** | 8 | 16 | 32 | 64 | 128 | 256 | 512 | 1024 |
| HB5 Δgp68 | *** | *** | *** | *** | *** | *** | ** | ** |
|  |  |  |  |  |  |  |  |  |
| **Figure 2C** | IVIG Dilution (1/X) | |  |  |  |  |  |  |
| **FcγRIIIA-ζ** | 8 | 16 | 32 | 64 | 128 | 256 | 512 | 1024 |
| HB5 ΔIRL Δgp34 | *** | *** | *** | *** | * | * | n.s. | n.s. |
| HB5 ΔIRL Δgp68/ Δgp34 | *** | *** | ** | * | n.s. | n.s. | n.s. | n.s. |
|  |  |  |  |  |  |  |  |  |
|  | IVIG Dilution (1/X) | |  |  |  |  |  |  |
| **FcγRIIA-ζ** | 8 | 16 | 32 | 64 | 128 | 256 | 512 | 1024 |
| HB5 ΔIRL Δgp34 | *** | *** | * | * | * | * | * | * |
| HB5 ΔIRL Δgp68/ Δgp34 | *** | *** | * | n.s. | n.s. | n.s. | n.s. | n.s. |
|  |  |  |  |  |  |  |  |  |
|  | IVIG Dilution (1/X) | |  |  |  |  |  |  |
| **FcγRI-ζ** | 8 | 16 | 32 | 64 | 128 | 256 | 512 | 1024 |
| HB5 ΔIRL Δgp34 | ** | *** | * | n.s. | n.s. | n.s. | n.s. | n.s. |
| HB5 ΔIRL Δgp68/ Δgp34 | *** | *** | ** | ** | n.s. | n.s. | n.s. | n.s. |
|  |  |  |  |  |  |  |  |  |
| **Figure 2D** | IVIG Dilution (1/X) | |  |  |  |  |  |  |
| **FcγRIIIA-ζ** | 8 | 16 | 32 | 64 | 128 | 256 | 512 | 1024 |
| AD169varL Δgp68 | *** | ** | *** | ** | * | n.s. | n.s. | n.s. |
| AD169varL Δgp34 | *** | ** | * | * | n.s. | n.s. | n.s. | n.s. |
| AD169varL Δgp68/ Δgp34 | *** | *** | *** | ** | ** | n.s. | n.s. | n.s. |
|  |  |  |  |  |  |  |  |  |
|  | IVIG Dilution (1/X) | |  |  |  |  |  |  |
| **FcγRIIA-ζ** | 8 | 16 | 32 | 64 | 128 | 256 | 512 | 1024 |
| AD169varL Δgp68 | *** | *** | *** | * | n.s. | n.s. | n.s. | n.s. |
| AD169varL Δgp34 | *** | *** | * | n.s. | n.s. | n.s. | n.s. | n.s. |
| AD169varL Δgp68/ Δgp34 | ** | ** | * | n.s. | n.s. | n.s. | n.s. | n.s. |
|  |  |  |  |  |  |  |  |  |
|  | IVIG Dilution (1/X) | |  |  |  |  |  |  |
| **FcγRI-ζ** | 8 | 16 | 32 | 64 | 128 | 256 | 512 | 1024 |
| AD169varL Δgp68 | *** | *** | *** | *** | *** | *** | ** | ** |
| AD169varL Δgp34 | ** | ** | *** | *** | *** | ** | ** | n.s. |
| AD169varL Δgp68/ Δgp34 | n.s. | n.s. | n.s. | *** | *** | * | n.s. | n.s. |
|  |  |  |  |  |  |  |  |  |
| **Figure 3A** | Herceptin Dilution (1/X) | |  |  |  |  |  |  |
| **FcγRIIIA-ζ** | 10 | 20 | 40 | 80 | 160 | 320 | 640 |  |
| rVACV gE | ** | ** | ** | ** | ** | * | * |  |
|  |  |  |  |  |  |  |  |  |
|  | Herceptin Dilution (1/X) | |  |  |  |  |  |  |
| **FcγRIIA-ζ** | 10 | 20 | 40 | 80 | 160 | 320 | 640 |  |
| rVACV gE | n.a. | n.a. | n.a. | n.a. | n.a. | n.a. | n.a. |  |
|  |  |  |  |  |  |  |  |  |
|  | Herceptin Dilution (1/X) | |  |  |  |  |  |  |
| **FcγRI-ζ** | 10 | 20 | 40 | 80 | 160 | 320 | 640 |  |
| rVACV gE | ** | *** | *** | ** | * | n.s. | n.s. |  |
|  |  |  |  |  |  |  |  |  |
| **Figure 3B** | Herceptin Dilution (1/X) | |  |  |  |  |  |  |
| **FcγRIIIA-ζ** | 10 | 20 | 40 | 80 | 160 | 320 | 640 |  |
| rVACV gp68 | *** | *** | ** | *** | *** | *** | *** |  |
| rVACV gp34 | *** | *** | *** | *** | *** | *** | *** |  |
|  |  |  |  |  |  |  |  |  |
|  | Herceptin Dilution (1/X) | |  |  |  |  |  |  |
| **FcγRIIA-ζ** | 10 | 20 | 40 | 80 | 160 | 320 | 640 |  |
| rVACV gp68 | n.a. | n.a. | n.a. | n.a. | n.a. | n.a. | n.a. |  |
| rVACV gp34 | n.a. | n.a. | n.a. | n.a. | n.a. | n.a. | n.a. |  |
|  |  |  |  |  |  |  |  |  |
|  | Herceptin Dilution (1/X) | |  |  |  |  |  |  |
|  | 10 | 20 | 40 | 80 | 160 | 320 | 640 |  |
| rVACV gp68 | *** | *** | *** | *** | *** | *** | *** |  |
| rVACV gp34 | *** | *** | *** | *** | *** | *** | *** |  |
|  |  |  |  |  |  |  |  |  |
| **Figure 4** | IgG1 | IgG2 | IgG3 | IgG4 | IgA | w/o Ab |  |  |
| rVACV gp68 | *** | n.a. | *** | n.s. | n.a. | n.a. |  |  |
| rVACV gp34 | *** | n.a. | *** | n.s. | n.a. | n.a. |  |  |
| rVACV MULT | n.s. | n.a. | n.s. | n.s. | n.a. | n.a. |  |  |
|  |  |  |  |  |  |  |  |  |
|  |  |  |  |  |  |  |  |  |
| **Figure 7A** | 1x | 5x | 10x | 50x |  |  |  |  |
| sgp34 | n.s | *** | ** | ** |  |  |  |  |
| sgp68 | n.s | * | *** | ** |  |  |  |  |
|  |  |  |  |  |  |  |  |  |
| **Figure 7B** | 1x | 10x | 50x |  |  |  |  |  |
| sgp34 | * | *** | ** |  |  |  |  |  |
| sgp68 | n.s. | n.s. | n.s. |  |  |  |  |  |
|  |  |  |  |  |  |  |  |  |
| **Figure 7C** | 1x | 10x | 75x |  |  |  |  |  |
| sgp34 | n.s. | *** | *** |  |  |  |  |  |
| sgp68 | n.s. | n.s. | ** |  |  |  |  |  |
|  |  |  |  |  |  |  |  |  |
| **Figure 7D** | 1x | 10x | 75x |  |  |  |  |  |
| sgp34 | n.s. | * | ** |  |  |  |  |  |
| sgp68 | n.s. | n.s. | * |  |  |  |  |  |
|  |  |  |  |  |  |  |  |  |
| **Figure 8A** | 1x | 5x | 20x | 50x |  |  |  |  |
| sgp34 | n.s. | *** | not measured | not measured |  |  |  |  |
| sgp68 | n.s. | * | * | *** |  |  |  |  |
|  |  |  |  |  |  |  |  |  |
| **Figure S4A** | 4 μg Rituximab |  |  |  |  |  |  |  |
| rVACV gE | * |  |  |  |  |  |  |  |
|  |  |  |  |  |  |  |  |  |
| **Figure S4B** | 4 μg Rituximab |  |  |  |  |  |  |  |
| rVACV gp68 | * |  |  |  |  |  |  |  |
| rVACV gp34 | * |  |  |  |  |  |  |  |
