# Supplementary material for: Human Cytomegalovirus Fcγ Binding Proteins gp34 and gp68 Antagonize Fcγ Receptors I, II and III
Source: PLoS Pathog. 2014 May 15;10(5):e1004131. doi: 10.1371/journal.ppat.1004131 (PMC4022731; doi:10.1371/journal.ppat.1004131)
Supplement: Table S2 — Synopsis of HCMV mutants used in the study. (DOCX) [file ppat.1004131.s007.docx]

Table S2

| Mutant | Primer | Primer sequence | Deletion (nt) | Deletion (aa) | Reference |
| --- | --- | --- | --- | --- | --- |
| HB5-ΔIRL | AZ-dIRL-1  AZ-dIRL-2  templ: HB5 | 5´GCGTCCAGTTCATGTAAAAGTCGGTCTCGCCGTGTCCGGCCACGAAGAGGCTGCTTACTACGATTTATTCAACAAAGCCACG  5´GGGGATCATTTATGGGGTCACCGCGTTGTTCGCGAAACATGAACTCCCACTGCCCGTGCGGCCAGTGTTACAACCAATTAACC | IRL:179150-192329 |  | Atalay et al., 2002 |
| HB5-ΔIRL/ΔTRL11 = Δgp34 | AZ-TRL11-tet1  AZ-TRL11-tet2  templ: HB5-ΔILR | 5´ACAGACGACGAAGAGGACGAGGACGACAACGTCTGATAAGGAAGGCGAGAACGTGTTTTGTCCAGTGAATTCGAGCTCGGTAC  5´TGTATACGCCGTATGCCTGTACGTGAGATGGTGAGGTCTTCGGCAGGCGACACGCATCTTGACCATGATTACGCCAAGCTCC | TRL11: 8722-9430  IRL: 179150-192329 | gp34 (1-234 | this study |
| HB5-ΔIRL/ΔTRL11/ΔUL118  = Δgp34/Δgp68 | AZ-TRL11-tet1  AZ-TRL11-tet2  templ: HB5-ΔILR/UL118 | 5´ACAGACGACGAAGAGGACGAGGACGACAACGTCTGATAAGGAAGGCGAGAACGTGTTTTGTCCAGTGAATTCGAGCTCGGTAC  5´TGTATACGCCGTATGCCTGTACGTGAGATGGTGAGGTCTTCGGCAGGCGACACGCATCTTGACCATGATTACGCCAAGCTCC | TRL11: 8722-9430  UL118:167913-168402  IRL: 179150 - 192329 | gp34 (1-234)  gp68 (143-345) | Halenius et al., 2011 |
| HB5-ΔUL118-120  = Δgp68 | AZ-UL118-120-1:  AZ- UL118-120-2:  templ: HB5 | 5´TGAGCAGTAGCGTTGTAGGAGAGATGTAGTTTTCCTGTGGATAAAATTCATAAGTTGTTTCGATTTATTCAACAAAGCCACG  5´AAAGCTATAAATCATAGCCACTGACGCAGGCACGTCATTGACAGCCGGCGACGTCGAGAGGCCAGTGTTACAACCAATTAACC | UL118-120:  168430- 169579 | gp68 (1-345) | Atalay et al., 2002 |
| pAD169-ΔUL119  = Δgp68 | KL-DeltaUL119-Kana1  KL-DeltaUL119-Kana2 | 5´TTGTTTATTTTGTTGGCAGGTTGGCGGGGGAGGAAAAGGGGTTGAACAGAAAGGTAGGTGCCAGTGAATTCGAGCTCGGTAC  5´AGGTGACGCGACCTCCTGCCACATATAGCTCGTCCACACGCCGTCTCGTCACACGGCAACGACCATGATTACGCCAAGCTCC | UL119: 168509-168939  (GenBank:FJ527563.1) | gp68 | this study |
| pAD169-ΔTRL11  = Δgp34 | KL-DeltaTRL11-Kana1  KL-DeltaTRL11-Kana2 | 5´ACGACGAAGAGGACGAGGACGACAACGTCTGATAAGGAAGGCGAGAACGTGTTTTGCACCCCAGTGAATTCGAGCTCGGTAC  5´TGTATACGCCGTATGCCTGTACGTGAGATGGTGAGGTCTTCGGCAGGCGACACGCATCTTGACCATGATTACGCCAAGCTCC | TRL11: 8750-9456  (GenBank:FJ527563.1) | gp34 | this study |
| pAD169-ΔTRL11/ΔUL119  = Δgp68/Δgp34 | KL-DeltaTRL11-Kana1  KL-DeltaTRL11-Kana2  KL-DeltaUL119-Kana1  KL-DeltaUL119-Kana2 |  | TRL11: 8750-9456  UL119: 168509-168939 | gp34  gp68 | this study |
| pTB40-ΔUL119  = Δgp68 | PL-TB40-UL119-tet1  PL-TB40-UL119-tet2  Temp: TB40 | 5´GGTCTCCTGCGGCCTGAGTCCCGAGATAAGCAGCTCTTGAGCAGTAGCGTTGTAGGAGAGccagtgaattcgagctcggtac  5´aggtgacgcgacctcctgccacatatagctcgtccacacgccgtctcgtcacacggcaacgaccatgattacgccaagctcc | 168527- 169046 | gp68 (1-142) | this study |
| pTB40-ΔTRL11  = Δgp34 | PL-TB40-TRL11-tet1  PL-TB40-TRL11-tet2  Temp: TB40 | 5´tccccgttgatcgaaccgacgggcacagacgacgaagaggacgaggacgacgacgtctgaccagtgaattcgagctcggtac  5´CATGCATGTTATTTGCGTGTACGATGACTTGTTTCGCCGTCGATGTTGTGTACGCATCTTACCATGATTACGCCAAGCTCC | 8826- 9558 | gp34 (1-234) | this study |
